# Supplementary material for: Derivation and validation of the BIMAST score for predicting the presence of fibrosis due to Metabolic dysfunction-associated steatotic liver disease among diabetic patients in the community
Source: PLoS One. 2024 Sep 27;19(9):e0307500. doi: 10.1371/journal.pone.0307500 (PMC11432895; doi:10.1371/journal.pone.0307500)
Supplement: S1 Appendix — (DOCX) [file pone.0307500.s001.docx]

**SUPPLEMENTARY MATERIAL**

**SUPPLEMENTARY METHODS**

**Screening procedures**

All patients were screened for liver disease and the presence of NAFLD with imaging ultrasound (US), blood tests (ferritin, anti-hepatitis C virus antibodies, hepatitis B virus serologic panel, auto-antibodies, ferritin, caeruloplasmin, alpha-1 antitrypsin and autoimmune profile) and transient elastography (TE). Other non-invasive markers of fibrosis (such as NAFLD fibrosis score and FIB-4) were calculated based on clinical parameters [1, 2], and ELF was obtained for each patient (The Doctors Laboratory, London, UK). Medical history, social history, alcohol consumption, dietary intake assessment and anthropometric parameters (Body Mass Index (BMI), waist and hip circumference) were also recorded for each patient. The patients’ ethnic backgrounds were as White Caucasian, White Hispanic, South Asian, East Asian, Black African or Afro-Caribbean, and Arab. Patients’ postcodes were used to assign a deprivation rank according to the English Index of Multiple Deprivation (IMD), as an estimation of socio-economic status.

TE and US were performed by a single operator, after fasting for 4 hours. Liver stiffness measurement (LSM) and controlled attenuation parameters (CAP) scores were both recorded. Only LSM meeting published criteria were included in the analysis [3]. The presence of hepatic steatosis on US was defined as per previously published criteria [4]. MASLD was defined as presence of steatosis with at least one cardiometabolic risk factor and in absence of other causes of liver disease, such as alcohol consumption > 14 IU/week and use of steatogenic medications. MASLD and increased alcohol intake (MetALD) was diagnosed in MASLD patients with chronic alcohol consumption > 14 IU per week[5]. Significant fibrosis was defined as LSM ≥ 8.1 kPa [6], while advanced fibrosis as LSM≥12.1 kPa [7]. A diagnosis of cirrhosis was based on histology or on a combination of imaging (irregular hepatic profile, caudate hypertrophy, splenomegaly) and biochemical features [7].

**TABLES**

| **Input parameter** | **Base-case value** | | **Ranges and probability distributions** | **Reference** | |
| --- | --- | --- | --- | --- | --- |
| **I *Transition probabilities*** | | | | | |
| **MLD to SLD** | 0.06-0.17 | |  | | Tanajewski, et al. |
| **SLD to CC** | 0.06-0.17 | | Dirichlet and Beta | | Tanajewski, et al. |
| **CC to DC** | 0.0735 | | Dirichlet and Beta | | Tanajewski, et al. |
| **CC to HCC** | 0.03 | | Dirichlet and Beta | | Tanajewski, et al. |
| **DC to HCC**  **<70 years**  **≥70 years** | 0.05  0.00 | | Dirichlet and Beta | | Tanajewski, et al. |
| **HCC to LT**  **<65 years**  **≥65 years** | 0.04  0.00 | |  | | Tanajewski, et al. |
| **All-cause mortality^1^**  **MLD to death**  **SLD to death**  **CC to death**  **DC to death**  **HCC to death**  **LT to death** | 0.019  0.019  0.0705  0.2275  0.133-0.53  0.029-0.166 | | Dirichlet and Beta | | Srivastava, et al. |
| **Risk ratio for diagnosed versus undiagnosed**  **MLD and SLD**  **CC** | 0.63  0.67 | | Log-normal  95% CI: 0.06-6.45  95% CI: 0.21-2.07 | | Tanajewski, et al. |
| **II *NAFLD screening*** | | 20, 40, 60, 80, 100 | | | |
| **Standard of care**  **mild liver disease (specificity^2^), %**  **Severe liver disease (sensitivity^2^), %**  **FIB-4,**  **No/mild liver disease (specificity), %**  **Severe liver disease (sensitivity), %**  **NAFLD fibrosis score**  **No/mild liver disease (specificity), %**  **Severe liver disease (sensitivity), %**  **TE**  **No/mild liver disease (specificity), %**  **Severe liver disease (sensitivity), %** | 65.00  35.00  83.65  62.75  62.68  62.00  21.63  98.04  100.00  100.00 | |  | | Srivastava, et al.  Primary care cohort  Primary care cohort  Primary care cohort |
| **III *Health state utilities*** | | Gamma, ±50% | | | |
| **MLD, SLD, CC**  **DC**  **HCC**  **LT**  **Death** | 0.895  0.66  0.65  0.69  0.00 | |  | | Tanajewski, et al. |
| **IV *Costs^3^ (2020 GBP)*** | | Gamma, ±50%, ±200% | | | |
| **Direct medical costs (standard of care strategy), first/subsequent year**  **MLD-**  **MLD+**  **False negatives**  **SLD+**  **CC**  **DC**  **HCC**  **LT**  **Direct medical costs (screening strategies), first/subsequent years**  **MLD-**  **MLD+**  **False negatives**  **SLD+**  **CC**  **DC**  **HCC**  **LT**  **Screening test unit costs**  **FIB-4**  **NAFLD fibrosis score**  **BIMAST**  **ELF**  **TE** | 0/0  1,400/74  0/0  1,400/421  1,975/1,012  7,639/8,823  22,228/20,806  102,223/23,686  0/0  171/171  0/0  805/605  1,179/1,179  7,639/8,823  22,228/20,806  102,223/23,686  4.24  4.72  0.89  66.04  125.00 | |  | | Tanajewski, et al.  Tanajewski, et al.  Primary care cohort |
| **V Other input parameters** |  | |  | |  |
| **Initial age, years**  **Discount rate for benefits and costs, %**  **Time horizon, years**  **NAFLD population prevalence, %** | 61  3.5  40 (lifetime)  64 | | 54-66  0-5  1, 5, 10, 20 | | Primary care cohort  NICE guidelines  Tanajewski, Srivastava  Primary care cohort |

^1^Adjusted by excess death rate due to diabetes from Tanajewski, et al.

^2^ Sensitivity=true positives/(true positives+false negatives); specificity=true negatives/(true negatives+false positives)

^3^ Direct medical costs include GP consultations, specialist and dietician appointments, medications and treatments associated with

each liver disease stage. Detailed cost components are reported in supplementary table 5.

**Supplementary table 1.** Model input parameters for the cost-effectiveness analysis of 5 NAFLD screening strategies compared to the standard of care, with data sources and ranges used in sensitivity analyses.

Abbreviations: MLD: mild liver disease (no MASLD or NAFLD with LSM≤8 kPa); SLD: significant liver disease (MASLD with LSM≥8.1 kPa); CC: compensated cirrhosis; False negatives: undiagnosed liver disease (MASLD with LSM ≥8.1 kPa who were false negatives at screening); DC: decompensated cirrhosis; HCC: hepatocellular carcinoma; TE: transient elastography.

| **Input parameter** | **Value (2020 GBP)** | **Reference** |
| --- | --- | --- |
| **I Annual direct medical costs: first year** | |  |
| **MLD**  FIB-4  GP appointment  Dietician appointment  Medications  **TOTAL** | 4.24  53.00  92.00  22.00  **171.24** | Tanajewski, et al. |
| **SLD**  Follow-up tests (TE, ultrasound)  GP appointment  1st referral to NAFLD clinic  Dietician appointment  Medications  **TOTAL** | 438.48  53.00  200.00  92.00  22.00  **805.48** | Tanajewski, et al. |
| **CC**  Follow-up tests (ultrasound, EEG, OGD)  1st referral to NAFLD clinic  Dietician appointment  Medications  **TOTAL** | 900.57  200.00  92.00  134.00  **1,179.57** | Tanajewski, et al. |
| **DC**  **HCC**  **Liver transplant** | 7,639.00  22,228.00  102,223.00 | Tanajewski, et al. |
| **II Annual direct medical costs: subsequent years** | | |
| **MLD**  FIB-4  GP appointment  Dietician appointment  Medications  **TOTAL** | 4.24  53.00  92.00  22.00  **171.24** | Tanajewski, et al. |
| **SLD**  Follow-up tests (TE, ultrasound)  Follow-up NAFLD clinic  Dietician appointment  Medications  **TOTAL** | 438.48  200.00  92.00  22.00  **605.48** | Tanajewski, et al. |
| **CC**  Follow-up tests (ultrasound, EEG, OGD)  Follow-up NAFLD clinic  Dietician appointment  Medications  **TOTAL** | 900.57  200.00  92.00  134.00  **1,179.57** | Tanajewski, et al. |
| **DC**  **HCC**  **Liver transplant** | 8,823.00  20,806.00  23,686.00 | Tanajewski, et al. |

**Supplementary table 2.** Comprehensive overview of unit costs by health state, used to calculate direct medical costs for screening strategies.

Abbreviations: MLD: mild liver disease (no MASLD or MASLD with LSM≤8 kPa); SLD: significant liver disease (MASLD with LSM≥8.1 kPa); CC: compensated cirrhosis; False negatives: undiagnosed liver disease (MASLD with LSM ≥8.1 kPa who were false negatives at screening); DC: decompensated cirrhosis; HCC: hepatocellular carcinoma; GP: general practitioner; EEG: electroencephalography; OGD: oesophago-gastro-duodenoscopy; TE: transient elastography.

| **Value of parameter varied** | **MASLD testing strategy** | **Discounted QALYs per person (years)** | **Discounted lifetime cost per person (£)** | **% change in correct diagnoses compared to baseline screening** | **ICER**  **(£/YLS)** |
| --- | --- | --- | --- | --- | --- |
| ***Base case (scenario 1)*** | | | | | |
| -- | Baseline | 12.29 | 12,295 | -- | -- |
|  | Screening | 12.89 | 13,543 | 30.41% | **£2,338** |
| ***Parameter varied: risk ratio (for diagnosed compared to undiagnosed)*** | | | | | |
| lower bound | Baseline | 13.68 | 5,627 | -- | -- |
|  | Screening | 14.10 | 5,783 | 30.41% | **£371** |
| upper bound | Baseline | 9.38 | 24,351 | -- | -- |
|  | Screening | 9.39 | 27,312 | 30.41% | **£799,213** |
| ***Parameter varied: time horizon*** | | | | | |
| 5 years | Baseline | 3.81 | 1,300 | -- | **--** |
|  | Screening | 3.82 | 1,599 | 30.41% | **£70,351** |
| 10 years | Baseline | 6.67 | 2,513 | -- | -- |
|  | Screening | 6.71 | 2,820 | 30.41% | **£7,536** |
| ***Parameter varied: health state utilities*** | | | | | |
| lower bound | Baseline | 11.98 | 12,295 | -- | -- |
|  | Screening | 12.49 | 13,543 | 30.41% | **£2,412** |
| upper bound | Baseline | 12.60 | 12,295 | -- | -- |
|  | Screening | 13.15 | 13,543 | 30.41% | **£2,268** |
| ***Parameter varied: direct medical costs*** | | | | | |
| 50% | Baseline | 12.29 | 6,148 | -- | -- |
|  | Screening | 12.82 | 6,771 | 30.41% | **£1,169** |
| 200% | Baseline | 12.29 | 24,591 | -- | -- |
|  | Screening | 12.82 | 27,086 | 30.41% | **£4,676** |
| ***Parameter varied: screening sensitivity and specificity*** | | | | | |
| 20% | Baseline | 12.29 | 12,295 | -- | -- |
|  | Screening | 12.59 | 13,265 | -68.34% | **£3,257** |
| 40% | Baseline | 12.29 | 12,295 | -- | -- |
|  | Screening | 12.73 | 13,221 | -36.67% | **£2,076** |
| 60% | Baseline | 12.29 | 12,295 | -- | -- |
|  | Screening | 12.80 | 13,341 | -5.01% | **£2,040** |
| 80% | Baseline | 12.29 | 12,295 | -- | -- |
|  | Screening | 12.84 | 13,516 | 26.65% | **£2,220** |
| 100% | Baseline | 12.29 | 12,295 | -- | -- |
|  | Screening | 12.86 | 13,718 | 58.31% | **£2,477** |
| ***Parameter varied: discount rate*** | | | | | |
| 1% | Baseline | 16.80 | 20,733 | -- | -- |
|  | Screening | 17.76 | 22,995 | 30.41% | **£2,353** |
| 5% | Baseline | 10.47 | 9,325 | -- | -- |
|  | Screening | 10.85 | 10,248 | 30.41% | **£2,393** |

**Supplementary table 3.** Results of the univariate sensitivity analyses showing the impact of uncertainty in model input parameters on the clinical outcomes and ICER: scenario 1 (US + LFTs).

| **Value of parameter varied** | **NAFLD screening** | **Discounted QALYs per person (years)** | **Discounted lifetime cost per person (£)** | **% change in correct diagnoses compared to baseline screening** | **ICER**  **(£/YLS)** |
| --- | --- | --- | --- | --- | --- |

| **Value of parameter varied** | **MASLD screening** | **Discounted QALYs per person (years)** | **Discounted lifetime cost per person (£)** | **% change in correct diagnoses compared to baseline screening** | **ICER**  **(£/YLS)** |
| --- | --- | --- | --- | --- | --- |
| ***Base case (scenario 2)*** | | | | | |
| -- | Baseline | 12.29 | 12,295 | -- | -- |
|  | Screening | 12.81 | 13,362 | 1.66% | **£2,060** |
| ***Parameter varied: risk ratio (for diagnosed compared to undiagnosed)*** | | | | | |
| lower bound | Baseline | 13.68 | 5,627 | -- | -- |
|  | Screening | 14.08 | 5,714 | -0.83% | **£215** |
| upper bound | Baseline | 12.60 | 12,295 | -- | -- |
|  | Screening | 13.14 | 13,362 | 1.66% | **£1,994** |
| ***Parameter varied: time horizon*** | | | | | |
| 5 years | Baseline | 3.81 | 1,300 | -- | **--** |
|  | Screening | 3.82 | 1,335 | 1.66% | **£8,350** |
| 10 years | Baseline | 6.67 | 2,513 | -- | -- |
|  | Screening | 6.71 | 2,581 | 1.66% | **£1,718** |
| ***Parameter varied: health state utilities*** | | | | | |
| lower bound | Baseline | 11.98 | 12,295 | -- | -- |
|  | Screening | 12.48 | 13,362 | 1.66% | **£2,130** |
| upper bound | Baseline | 12.60 | 12,295 | -- | -- |
|  | Screening | 13.14 | 13,362 | 1.66% | **£1,994** |
| ***Parameter varied: direct medical costs*** | | | | | |
| 50% | Baseline | 12.29 | 6,148 | -- | -- |
|  | Screening | 12.81 | 6,681 | 1.66% | **£1,030** |
| 200% | Baseline | 12.29 | 24,591 | -- | -- |
|  | Screening | 12.81 | 26,724 | 1.66% | **£4,120** |
| ***Parameter varied: screening sensitivity and specificity*** | | | | | |
| 20% | Baseline | 12.29 | 12,295 | -- | -- |
|  | Screening | 12.59 | 13,265 | -68.34% | **£3,257** |
| 40% | Baseline | 12.29 | 12,295 | -- | -- |
|  | Screening | 12.73 | 13,221 | -36.67% | **£2,076** |
| 60% | Baseline | 12.29 | 12,295 | -- | -- |
|  | Screening | 12.80 | 13,341 | -5.01% | **£2,040** |
| 80% | Baseline | 12.29 | 12,295 | -- | -- |
|  | Screening | 12.84 | 13,516 | 26.65% | **£2,220** |
| 100% | Baseline | 12.29 | 12,295 | -- | -- |
|  | Screening | 12.86 | 13,718 | 58.31% | **£2,477** |
| ***Parameter varied: discount rate*** | | | | | |
| 1% | Baseline | 16.80 | 20,733 | -- | -- |
|  | Screening | 17.73 | 22,841 | 1.66% | **£2,259** |
| 5% | Baseline | 10.47 | 9,325 | -- | -- |
|  | Screening | 10.84 | 10,055 | 30.41% | **£1,953** |

**Supplementary table 4.** Results of the univariate sensitivity analyses showing the impact of uncertainty in model input parameters on the clinical outcomes and ICER: scenario 2 (FIB-4).

Abbreviations: ICER: incremental cost-effectiveness ratio; QALY: quality-adjusted year.

| **Value of parameter varied** | **MASLD screening** | **Discounted QALYs per person (years)** | **Discounted lifetime cost per person (£)** | **% change in correct diagnoses compared to baseline screening** | **ICER**  **(£/YLS)** |
| --- | --- | --- | --- | --- | --- |
| ***Base case (scenario 3)*** | | | | | |
| -- | Baseline | 12.29 | 12,295 | -- | -- |
|  | Screening | 12.76 | 13,275 | -58.36% | **£2,092** |
| ***Parameter varied: risk ratio (for diagnosed compared to undiagnosed)*** | | | | | |
| lower bound | Baseline | 13.68 | 5,627 | -- | -- |
|  | Screening | 14.08 | 5,960 | -58.36% | **£827** |
| upper bound | Baseline | 9.39 | 24,351 | -- | -- |
|  | Screening | 9.59 | 25,558 | -58.36% | **£6,016** |
| ***Parameter varied: time horizon*** | | | | | |
| 5 years | Baseline | 3.81 | 1,300 | -- | **--** |
|  | Screening | 3.82 | 830 | -58.36% | **-£109,221** |
| 10 years | Baseline | 6.67 | 2,513 | -- | -- |
|  | Screening | 6.70 | 2,160 | -58.36% | **-£9,080** |
| ***Parameter varied: health state utilities*** | | | | | |
| lower bound | Baseline | 11.98 | 12,295 | -- | -- |
|  | Screening | 12.42 | 13,275 | -58.36% | **£2,184** |
| upper bound | Baseline | 12.60 | 12,295 | -- | -- |
|  | Screening | 13.09 | 13,275 | -58.36% | **£2,008** |
| ***Parameter varied: direct medical costs*** | | | | | |
| 50% | Baseline | 12.29 | 6,148 | -- | -- |
|  | Screening | 12.76 | 6,638 | -58.36% | **£1,046** |
| 200% | Baseline | 12.29 | 24,591 | -- | -- |
|  | Screening | 12.76 | 26,550 | 30.41% | **£4,185** |
| ***Parameter varied: screening sensitivity and specificity*** | | | | | |
| 20% | Baseline | 12.29 | 12,295 | -- | -- |
|  | Screening | 12.59 | 13,265 | -68.34% | **£3,257** |
| 40% | Baseline | 12.29 | 12,295 | -- | -- |
|  | Screening | 12.73 | 13,221 | -36.67% | **£2,076** |
| 60% | Baseline | 12.29 | 12,295 | -- | -- |
|  | Screening | 12.80 | 13,341 | -5.01% | **£2,040** |
| 80% | Baseline | 12.29 | 12,295 | -- | -- |
|  | Screening | 12.84 | 13,516 | 26.65% | **£2,220** |
| 100% | Baseline | 12.29 | 12,295 | -- | -- |
|  | Screening | 12.86 | 13,718 | 58.31% | **£2,477** |
| ***Parameter varied: discount rate*** | | | | | |
| 1% | Baseline | 16.80 | 20,733 | -- | -- |
|  | Screening | 17.64 | 22,948 | 58.31% | **£2,636** |
| 5% | Baseline | 10.47 | 9,325 | -- | -- |
|  | Screening | 10.80 | 9,891 | 30.41% | **£1,669** |

**Supplementary table 5.** Results of the univariate sensitivity analyses showing the impact of uncertainty in model input parameters on the clinical outcomes and ICER: scenario 3 (NAFLD fibrosis score).

Abbreviations: ICER: incremental cost-effectiveness ratio; QALY: quality-adjusted year.

| **Value of parameter varied** | **MASLD screening** | **Discounted QALYs per person (years)** | **Discounted lifetime cost per person (£)** | **% change in correct diagnoses compared to baseline screening** | **ICER**  **(£/YLS)** |
| --- | --- | --- | --- | --- | --- |
| ***Base case (scenario 4)*** | | | | | |
| -- | Baseline | 12.29 | 12,295 | -- | -- |
|  | Screening | 12.83 | 13,366 | 10.76% | **£1,967** |
| ***Parameter varied: risk ratio (for diagnosed compared to undiagnosed)*** | | | | | |
| lower bound | Baseline | 13.68 | 5,627 | -- | -- |
|  | Screening | 14.14 | 5,545 | 7.96% | **-£176** |
| upper bound | Baseline | 9.38 | 24,351 | -- | -- |
|  | Screening | 9.32 | 27,366 | 4.86% | **-£46,796** |
| ***Parameter varied: time horizon*** | | | | | |
| 5 years | Baseline | 3.81 | 1,300 | -- | **--** |
|  | Screening | 3.82 | 1,368 | 4.55% | **£15,368** |
| 10 years | Baseline | 6.67 | 2,513 | -- | -- |
|  | Screening | 6.71 | 2,614 | 6.03% | **£2,428** |
| ***Parameter varied: health state utilities*** | | | | | |
| lower bound | Baseline | 11.98 | 12,295 | -- | -- |
|  | Screening | 12.50 | 13,366 | 10.76% | **£2,027** |
| upper bound | Baseline | 12.60 | 12,295 | -- | -- |
|  | Screening | 13.16 | 13,366 | 10.76% | **£1,910** |
| ***Parameter varied: direct medical costs*** | | | | | |
| 50% | Baseline | 12.29 | 6,148 | -- | -- |
|  | Screening | 12.83 | 6,683 | 10.76% | **£984** |
| 200% | Baseline | 12.29 | 24,591 | -- | -- |
|  | Screening | 12.83 | 26,733 | 10.76% | **£3,934** |
| ***Parameter varied: screening sensitivity and specificity*** | | | | | |
| 20% | Baseline | 12.29 | 12,295 | -- | -- |
|  | Screening | 12.59 | 13,265 | -68.34% | **£3,257** |
| 40% | Baseline | 12.29 | 12,295 | -- | -- |
|  | Screening | 12.73 | 13,221 | -36.67% | **£2,076** |
| 60% | Baseline | 12.29 | 12,295 | -- | -- |
|  | Screening | 12.80 | 13,341 | -5.01% | **£2,040** |
| 80% | Baseline | 12.29 | 12,295 | -- | -- |
|  | Screening | 12.84 | 13,516 | 26.65% | **£2,220** |
| 100% | Baseline | 12.29 | 12,295 | -- | -- |
|  | Screening | 12.86 | 13,718 | 58.31% | **£2,477** |
| ***Parameter varied: discount rate*** | | | | | |
| 1% | Baseline | 16.80 | 20,733 | -- | -- |
|  | Screening | 17.78 | 22,837 | 10.76% | **£2,144** |
| 5% | Baseline | 10.47 | 9,325 | -- | -- |
|  | Screening | 10.86 | 10,065 | 10.76% | **£1,879** |

**Supplementary table 6.** Results of the univariate sensitivity analyses showing the impact of uncertainty in model input parameters on the clinical outcomes and ICER: scenario 4 (BIMAST).

| **Value of parameter varied** | **MASLD screening** | **Discounted QALYs per person (years)** | **Discounted lifetime cost per person (£)** | **% change in correct diagnoses compared to baseline screening** | **ICER**  **(£/YLS)** |  |
| --- | --- | --- | --- | --- | --- | --- |
| ***Base case (scenario 5)*** | | | | | |  |
| -- | Baseline | 12.29 | 12,296 | -- | -- |  |
|  | Screening | 12.80 | 13,588 | 8.48% | **£2,541** |  |
| ***Parameter varied: risk ratio (for diagnosed compared to undiagnosed)*** | | | | | |  |
| lower bound | Baseline | 13.68 | 5,627 | -- | -- |  |
|  | Screening | 14.05 | 5,980 | 6.20% | **£952** |  |
| upper bound | Baseline | 9.38 | 24,351 | -- | -- |  |
|  | Screening | 9.51 | 26,805 | 6.12% | **£19,510** |  |
| ***Parameter varied: time horizon*** | | | | | |  |
| 5 years | Baseline | 3.81 | 1,300 | -- | **--** |  |
|  | Screening | 3.82 | 1,630 | 11.21% | **£80,343** |  |
| 10 years | Baseline | 6.67 | 2,513 | -- | -- |  |
|  | Screening | 6.71 | 2,842 | 7.34% | **£8,410** |  |
| ***Parameter varied: health state utilities*** | | | | | |  |
| lower bound | Baseline | 11.98 | 12,295 | -- | -- |  |
|  | Screening | 12.47 | 13,588 | 8.48% | **£2,630** |  |
| upper bound | Baseline | 12.60 | 12,295 | -- | -- |  |
|  | Screening | 13.13 | 13,588 | 8.48% | **£2,458** |  |
| ***Parameter varied: direct medical costs*** | | | | | |  |
| 50% | Baseline | 12.29 | 6,148 | -- | -- |  |
|  | Screening | 12.80 | 6,794 | 8.48% | **£1,271** |  |
| 200% | Baseline | 12.29 | 24,591 | -- | -- |  |
|  | Screening | 12.80 | 27,175 | 8.48% | **£5,082** |  |
| ***Parameter varied: screening sensitivity and specificity*** | | | | | |  |
| 20% | Baseline | 12.29 | 12,295 | -- | -- |  |
|  | Screening | 12.59 | 13,265 | -19.20% | **£3,257** |  |
| 40% | Baseline | 12.29 | 12,295 | -- | -- |  |
|  | Screening | 12.73 | 13,221 | -49.67% | **£2,076** |  |
| 60% | Baseline | 12.29 | 12,295 | -- | -- |  |
|  | Screening | 12.80 | 13,341 | 7.63% | **£2,040** |  |
| 80% | Baseline | 12.29 | 12,295 | -- | -- |  |
|  | Screening | 12.84 | 13,516 | 12.16% | **£2,220** |  |
| 100% | Baseline | 12.29 | 12,295 | -- | -- |  |
|  | Screening | 12.86 | 13,718 | 15.05% | **£2,477** |  |
| ***Parameter varied: discount rate*** | | | | | |  |
| 1% | Baseline | 16.80 | 20,733 | -- | -- |  |
|  | Screening | 17.72 | 23,046 | 8.48% | **£2,526** |  |
| 5% | Baseline | 10.47 | 9,325 | -- | -- |  |
|  | Screening | 10.83 | 10,289 | 8.48% | | **£2,623** |

**Supplementary table 7.** Results of the univariate sensitivity analyses showing the impact of uncertainty in model input parameters on the clinical outcomes and ICER: scenario 4 (ELF).

Abbreviations: ICER: incremental cost-effectiveness ratio; QALY: quality-adjusted year.

| **Value of parameter varied** | **MASLD screening** | **Discounted QALYs per person (years)** | **Discounted lifetime cost per person (£)** | **% change in correct diagnoses compared to baseline screening** | **ICER**  **(£/YLS)** |
| --- | --- | --- | --- | --- | --- |
| ***Base case (scenario 6)*** | | | | | |
| -- | Baseline | 12.29 | 12,289 | -- | -- |
|  | Screening | 12.86 | 13,718 | 15.05% | **£2,478** |
| ***Parameter varied: risk ratio (for diagnosed compared to undiagnosed)*** | | | | | |
| lower bound | Baseline | 13.68 | 5,627 | -- | -- |
|  | Screening | 14.17 | 5,701 | 11.05% | **£149** |
| upper bound | Baseline | 9.38 | 24,351 | -- | -- |
|  | Screening | 9.21 | 28,442 | 10.43% | **-£23,192** |
| ***Parameter varied: time horizon*** | | | | | |
| 5 years | Baseline | 3.81 | 1,300 | -- | **--** |
|  | Screening | 3.82 | 1,854 | 18.96% | **£120,487** |
| 10 years | Baseline | 6.67 | 2,513 | -- | -- |
|  | Screening | 6.71 | 3,062 | 13.85% | **£12,441** |
| ***Parameter varied: health state utilities*** | | | | | |
| lower bound | Baseline | 11.98 | 12,295 | -- | -- |
|  | Screening | 12.54 | 13,718 | 15.05% | **£2,543** |
| upper bound | Baseline | 12.60 | 12,295 | -- | -- |
|  | Screening | 13.19 | 13,718 | 15.05% | **£2,413** |
| ***Parameter varied: direct medical costs*** | | | | | |
| 50% | Baseline | 12.29 | 6,148 | -- | -- |
|  | Screening | 12.86 | 6,859 | 15.05% | **£1,238** |
| 200% | Baseline | 12.29 | 24,591 | -- | -- |
|  | Screening | 12.86 | 27,435 | 15.05% | **£4,953** |
| ***Parameter varied: screening sensitivity and specificity*** | | | | | |
| 20% | Baseline | 12.29 | 12,295 | -- | -- |
|  | Screening | 12.59 | 13,265 | -68.34% | **£3,257** |
| 40% | Baseline | 12.29 | 12,295 | -- | -- |
|  | Screening | 12.73 | 13,221 | -36.67% | **£2,076** |
| 60% | Baseline | 12.29 | 12,295 | -- | -- |
|  | Screening | 12.80 | 13,341 | -5.01% | **£2,040** |
| 80% | Baseline | 12.29 | 12,295 | -- | -- |
|  | Screening | 12.84 | 13,516 | 26.65% | **£2,220** |
| 100% | Baseline | 12.29 | 12,295 | -- | -- |
|  | Screening | 12.86 | 13,718 | 58.31% | **£2,477** |
| ***Parameter varied: discount rate*** | | | | | |
| 1% | Baseline | 16.80 | 20,733 | -- | -- |
|  | Screening | 17.84 | 23,144 | 15.05% | **£2,332** |
| 5% | Baseline | 10.47 | 9,325 | -- | -- |
|  | Screening | 10.88 | 10,433 | 15.05% | **£2,670** |

**Supplementary table 8.** Results of the univariate sensitivity analyses showing the impact of uncertainty in model input parameters on the clinical outcomes and ICER: scenario 6 (TE).

Abbreviations: ICER: incremental cost-effectiveness ratio; QALY: quality-adjusted year.

|  | **Study population**  **N=287** | **MASLD**  **N=186** | **Normal liver**  **N=73** |  |
| --- | --- | --- | --- | --- |
|  | **Median (IQR)** | **Median (IQR)** | **Median (IQR)** | **P value*** |
| **Age,** *years* | 59 (59-66) | 60 (54-66) | 59 (53-65) | 0.83 |
| **Waist circum,** *cm* | 107 (107-116) | **108 (101-118)** | **98 (92-106)** | **0.0001** |
| **Hip circum,** *cm* | 110 (102-119) | **112 (105-122)** | **103 (98-108)** | **0.0001** |
| **BMI,** *kg/m^2^* | 30.8 (26.9-34.4) | **31.4 (28.4-35.8)** | **26.9 (24.8-30.3)** | **0.0001** |
| **PLT,** *x 10^9^/µL* | 250 (202-290) | 245 (212-287) | 249 (206-298) | 0.88 |
| **ALT,** *IU/L* | 35 (22-45) | **34 (23-49)** | **24 (18-28)** | **0.0001** |
| **AST,** *IU/L* | 31 (22-35) | **28 (23-37)** | **24 (19-27)** | **0.0001** |
| **GGT,** *IU/L* | 47 (19-50) | **32 (22-52)** | **19 (17-27)** | **0.0001** |
| **ALP,** *IU/L* | 88 (70-103) | 84 (72-105) | 85 (63-99) | 0.7 |
| **Albumin,** *g/L* | 40 (39-42) | 41 (39-42) | 40 (39-42) | 0.83 |
| **Bilirubin,** *µmol/L* | 10.6 (6-12) | 9 (6-12) | 8 (6-14) | 0.55 |
| **Total Cholesterol,** *mmol/l* | 4.1 (3.5-4.7) | 4.1 (3.4-4.7) | 4 (3.6-4.5) | 0.58 |
| **TRG,** *mmol/l* | 2.3 (1.02-2.08) | 1.4 (1.07-2.1) | 1.2 (0.98-1.5) | 0.25 |
| **HDL,** *mmol/l* | 1.1 (0.9-1.3) | 1.1 (0.9-1.2) | 1.16 (1.06-1.39) | 0.25 |
| **LDL,** *mmol/l* | 2.3 (1.6-2.7) | 2.2 (1.6-2.8) | 2.1 (1.7-2.6) | 0.68 |
| **Ferritin,** *ng/ml* | 124 (43-155) | 82 (39-140) | 70 (28-178) | 0.91 |
| **Diabetes characteristics** | | | | |
|  | **Median (IQR)** | **Median (IQR)** | **Median (IQR)** | **P value*** |
| **Fasting glucose,** *mmol/l* | 7.9 (5.5) | **7.4 (5.6-10.2)** | **6.2 (4.8-7.8)** | **0.001** |
| **HbA1c,** *mmol/mol* | 60 (49-70) | **60 (50-74)** | **55 (48-61)** | **0.0001** |
| **Insulin,** *µU/ml* | 24 (8.1-26.5) | **15.3 (9.8-28.2)** | **7.2 (5.8-12.2)** | **0.028** |
| **Homa index** | 8 (1.9-8.95) | **4.6 (2.2-10.3)** | **2.1 (1.35-4.8)** | **0.0001** |
| **Duration DM,** *years* | 11 (4-16) | 10 (3-16) | 13 (7-16) | 0.16 |
|  | **N (%)** | **N (%)** | **N (%)** | **P value*** |
| **Diet controlled** | 39 (13) | 25 (13) | 13 (18) | 0.11 |
| **On oral agents** | 227 (79) | 170 (91) | 55 (75) | 0.16 |
| **On GLP-1RA** | 37 (13) | 31 (16) | 6 (8) | 0.08 |
| **On insulin** | 74 (25) | 51 (28) | 23 (31) | 0.18 |
| **Diabetic complications** | 45 (16) | 26 (14) | 15 (21) | 0.82 |
| **Ethnic background and comorbidities** | | | | |
|  | **N (%)** | **N (%)** | **N (%)** | **P value*** |
| **Male gender** | 160 (53) | 104 (56) | 34 (45) | 0.07 |
| **White, Caucasian** | 102 (32) | **64 (34)** | **15 (20)** | **0.02** |
| **White, Hispanic** | 6 (2) | 3 (1) | 2 (2) | 0.43 |
| **Black African, Afro-Caribbean** | 33 (12) | 22 (12) | 10 (13) | 0.41 |
| **Arab** | 74 (28) | 52 (28) | 20 (26) | 0.52 |
| **South Asian** | 47 (17) | 31 (17) | 16 (21) | 0.2 |
| **East Asian** | 24 (8) | 14 (7) | 10 (13) | 0.09 |
| **Hypertension** | 191 (67) | 120 (64) | 50 (66) | 0.32 |
| **Dyslipidaemia** | 148 (52) | 98 (53) | 39 (52) | 0.51 |
| **Psychiatric disorder** | 41 (15) | 27 (14) | 11 (14) | 0.53 |
| **Previous ACE** | 28 (10) | 16 (8) | 11 (14) | 0.98 |
| **On statin** | 214 (75) | 138 (74) | 57 (76) | 0.31 |

**Supplementary table 9.** **Characteristics of the study population and differences between patients with and without MASLD.** The table shows the differences between patients with (n=186) and without (n=73) MASLD in the whole study population (n=287). Variables are expressed as median and IQR or relative percentages. * p-value refers to differences between patients with MASLD and normal liver.

*Abbreviations: IQR: interquartile range, BMI: Body mass index, PLT: platelet, ALT: alanine aminotransferase, AST: aspartate aminotransferase, GGT: gamma-glutamyl transferase, ALP: alkaline phosphatase, TRG: triglycerides, HDL: high density lipoprotein, LDL: low density lipoprotein, HbA1c: glycated haemoglobin, GLP-1RA: glucagon like peptide-1 receptor agonist*

|  | **MASLD,**  **LSM≥8.1 kPa**  **N=50** | **MASLD,**  **normal LSM**  **N=136** |  |
| --- | --- | --- | --- |
|  | **Median (IQR)** | **Median (IQR)** | **P value*** |
| **Age,** *years* | 60 (51-65) | 61 (54-65) | 0.49 |
| **Waist circum,** *cm* | **120 (112-127)** | **105 (99-113)** | **0.0001** |
| **Hip circum,** *cm* | **123 (123-132)** | **110 (103-119)** | **0.0001** |
| **BMI,** *kg/m^2^* | **36.8 (32-39.7)** | **30.3 (27.6-33.6)** | **0.0001** |
| **PLT,** *x 10^9^/uL* | 231 (198-266) | 255 (215-300) | 0.3 |
| **ALT,** *IU/L* | **46 (25-60)** | **30 (22-43)** | **0.0001** |
| **AST,** *IU/L* | **37 (28-48)** | **26 (22-32)** | **0.0001** |
| **GGT,** *IU/L* | **62 (35-96)** | **27 (19-39)** | **0.0001** |
| **ALP,** *IU/L* | 83 (70-110) | 84 (72-101) | 0.62 |
| **Albumin,** *g/L* | 40 (38-41) | 41 (39-42) | 0.06 |
| **Bilirubin,** *µmol/L* | 10 (7-16) | 8 (6-11) | 0.55 |
| **Total Cholesterol,** *mmol/l* | 3.9 (3.4-4.4) | 4.1 (3.5-4.8) | 0.14 |
| **TRG,** *mmol/l* | 1.3 (1.08-2.2) | 1.5 (1.06-2.1) | 0.92 |
| **HDL,** *mmol/l* | 1.1 (0.9-1.2) | 1.08 (0.9-1.3) | 0.51 |
| **LDL,** *mmol/l* | 1.9 (1.6-2.6) | 2.2 (1.6-2.8) | 0.42 |
| **Ferritin,** *ng/ml* | 108 (48-182) | 81 (36-124) | 0.31 |
| **Diabetes characteristics** | | | |
|  | **Median (IQR)** | **Median (IQR)** | **P value*** |
| **Fasting glucose,** *mmol/l* | **9.4 (6.2-13.4)** | **6.7 (5.2-9.2)** | **0.001** |
| **HbA1c,** *mmol/mol* | **71 (56-84)** | **59 (49-68)** | **0.0001** |
| **Insulin,** *µU/ml* | **21 (14-37.2)** | **12.4 (9-25)** | **0.001** |
| **Homa index** | **8.1 (4.5-14.1)** | **3.3 (2.1-8.4)** | **0.001** |
| **Duration DM,** *years* | 10 (4-16) | 10 (3-16) | 0.46 |
|  | **N (%)** | **N (%)** | **P value*** |
| **Diet controlled** | 1 (2) | 24 (17) | 0.052 |
| **On oral agents** | 43 (86) | 127 (93) | 0.051 |
| **On GLP-1-RA** | 10 (20) | 21 (15) | 0.07 |
| **On insulin** | 15 (30) | 36 (26) | 0.25 |
| **Diabetic complications** | 10 (20) | 16 (12) | 0.82 |
| **Ethnic background and comorbidities** | | | |
|  | **N (%)** | **N (%)** | **P value*** |
| **Male gender** | 29 (58) | 75 (55) | 0.44 |
| **White, Caucasian** | 20 (40) | 45 (33) | 0.22 |
| **White, Hispanic** | 1 (2) | 2 (1) | 0.61 |
| **Black African, Afro-Caribbean** | 4 (8) | 18 (13) | 0.23 |
| **Arab** | 15 (30) | 37 (27) | 0.43 |
| **South Asian** | 8 (16) | 22 (16) | 0.47 |
| **East Asian** | 2 (4) | 12 (9) | 0.21 |
| **Hypertension** | 33 (66) | 87 (63) | 0.45 |
| **Dyslipidaemia** | 27 (54) | 71 (52) | 0.46 |
| **Psychiatric disorder** | 9 (18) | 19 (13) | 0.28 |
| **Previous ACE** | 3 (6) | 13 (9) | 0.29 |
| **On statin** | 39 (78) | 99 (76) | 0.32 |

**Supplementary table 10.** **Differences between MASLD patients stratified per liver stiffness measurement greater than 8.1 kPa.** The table shows the differences between patients with MASLD with elevated (n=50) and normal (n=136) LSM. Variables are expressed as median and IQR or relative percentages. * p-value: differences between patients with MASLD with elevated LSM and normal LSM.

*Abbreviations: IQR: interquartile range, BMI: Body mass index, PLT: platelet, ALT: alanine aminotransferase, AST: aspartate aminotransferase, GGT: gamma-glutamyl transferase, ALP: alkaline phosphatase, TRG: triglycerides, HDL: high density lipoprotein, LDL: low density lipoprotein, HbA1c: glycated haemoglobin, GLP-1RA: glucagon like peptide-1 receptor agonist*

|  | **MASLD, elevated LSM**  **N=50** | **MASLD, normal LSM**  **N=136** |  |
| --- | --- | --- | --- |
|  | **Median (IQR)** | **Median (IQR)** | **P value*** |
| **Overall IMD** | 10043  (4098-18528) | 11858  (4851-21254) | 0.1 |
| **Income rank** | 6767  (1872-16991) | 8336  (3218-20084) | 0.19 |
| **Employment rank** | 8171  (3109-19674) | 10511  (3110-24241) | 0.22 |
| **Education rank** | **18789**  **(13721-26362)** | **23148**  **(14665-28792)** | **0.03** |
| **Health and disability rank** | 16806  (9800-27198) | 20105  (12063-29788) | 0.13 |
| **Crime rank** | 14692  (7268-20746) | 16118  (8228-22680) | 0.63 |
| **Barriers to housing services rank** | 11292  (8067-13945) | 11728  (9393-14434) | 0.29 |
| **Living environment rank** | 5923  (2769-9394) | 5599  (3289-8107) | 0.99 |
|  | **N (%)** | **N (%)** | **P value*** |
| **Overall IMD**  1^st^ tertile  3^rd^ tertile | 27 (54)  7 (14) | 56 (41)  29 (21) | 0.2 |
| **Income rank**  1^st^ tertile  3^rd^ tertile | 27 (54)  36 (51) | 73 (53)  27 (20) | 0.06 |
| **Employment rank**  1^st^ tertile  3^rd^ tertile | 27 (54)  9 (16) | 66 (48)  37 (27) | 0.42 |
| **Education rank**  1^st^ tertile  3^rd^ tertile | 6 (12)  18 (36) | 13 (10)  71 (52) | 0.08 |
| **Health and disability rank**  1^st^ tertile  3^rd^ tertile | 13 (25)  15 (30) | 21 (15)  61 (44) | 0.54 |
| **Crime rank**  1^st^ tertile  3^rd^ tertile | 17 (34)  10 (20) | 38 (27)  33 (24) | 0.71 |
| **Barriers to housing services rank**  1^st^ tertile  3^rd^ tertile | 18 (36)  0 (0) | 35 (25)  1 (0) | 0.65 |
| **Living environment rank**  1^st^ tertile  3^rd^ tertile | 38 (76)  1 (2) | 114 (83)  0 (0) | 0.73 |

**Supplementary table 11. Differences in socio-economic status between MASLD patients stratified per liver stiffness measurement greater than 8.1 kPa.** Socio-economic status is expressed as Index of multiple deprivation and relative single domains. The table shows the differences between patients with MASLD with elevated (n=50) and normal (n=136) LSM. Variables are expressed as median and IQR or relative percentages. * p-value: differences between patients with MASLD with elevated LSM and normal LSM.

*Abbreviations: MASLD: Metabolic-dysfunction associated steatotic liver disease, LSM: liver stiffness measurement, IQR: interquartile range, IMD: index of multiple deprivation.*

|  | **Derivation cohort**  **N=194** | **Validation cohort**  **N=93** |  |
| --- | --- | --- | --- |
|  | **Median (IQR)** | **Median (IQR)** | **P value*** |
| **Age,** *years* | 60 (54-66) | 61 (54-65) | 0.46 |
| **Waist circum,** *cm* | 106 (98-116) | 106 (100-107) | 0.38 |
| **Hip circum,** *cm* | 109 (102-118) | 112 (105-121) | 0.16 |
| **BMI,** *kg/m^2^* | 30 (26.7-34.4) | 31.1 (28.1-34.4) | 0.39 |
| **PLT,** *x 10^9^/uL* | 254 (212-292) | 234 (192-275) | 0.055 |
| **ALT,** *IU/L* | 28 (20-43) | 32 (26-48) | 0.07 |
| **AST,** *IU/L* | 27 (22-34) | 28 (23-39) | 0.18 |
| **GGT,** *IU/L* | 27 (18-46) | 31 (19-59) | 0.31 |
| **ALP,** *IU/L* | 83 (69-100) | 85 (23-39) | 0.1 |
| **Total Cholesterol,** *mmol/l* | 4.1 (3.5-4.7) | 4.1 (3.4-4.8) | 0.9 |
| **TRG,** *mmol/l* | 1.3 (1-2) | 1.4 (1-2) | 0.93 |
| **HDL,** *mmol/l* | 1.1 (0.9-1.3) | 1.1 (0.9-1.3) | 0.69 |
| **LDL,** *mmol/l* | 2.1 (1.2-2.7) | 2.1 (1.5-2.8) | 0.77 |
| **Ferritin,** *ng/ml* | 81 (40-155) | 108 (46-156) | 0.37 |
| **LSM,** *kPa* | 5.6 (4.4-7.3) | 5.5 (4-7.5) | 0.56 |
| **CAP score,** *dB/m* | 309 (255-292) | 308 (260-347) | 0.81 |
| **Diabetes characteristics** | | | |
|  | **Median (IQR)** | **Median (IQR)** | **P value*** |
| **Fasting glucose,** *mmol/l* | 6.8 (5.2-9.4) | 6.9 (5.8-9.9) | 0.07 |
| **HbA1c,** *mmol/mol* | 57 (49-70) | 59 (47-71) | 0.75 |
| **Insulin,** *µU/ml* | 14 (9-27) | 12.4 (7.4-22) | 0.17 |
| **Homa index** | 4.1 (2.1-8.4) | 3.2 (1.8-9.6) | 0.47 |
| **Duration DM,** *years* | 10 (4-18) | 10 (4-15) | 0.055 |

**Supplementary table 12. Differences between derivation and internal validation cohort.** The table shows the differences between patients from the validation vs internal derivation cohort. Variables are expressed as median and IQR or relative percentages.

* p-value: differences between patients from the derivation vs internal validation cohort.

*Abbreviations: IQR: interquartile range, BMI: Body mass index, PLT: platelet, ALT: alanine aminotransferase, AST: aspartate aminotransferase, GGT: gamma-glutamyl transferase, ALP: alkaline phosphatase, TRG: triglycerides, HDL: high density lipoprotein, LDL: low density lipoprotein, HbA1c: glycated haemoglobin.*

| **Variable** | **Sig.** | **Crude OR** | **95% CI** | |
| --- | --- | --- | --- | --- |
|  |  |  | **Lower** | **Upper** |
| **Waist circumference,** *cm* | **0.008** | **1.086** | **1.021** | **1.154** |
| **Hip circumference,** *cm* | 0.659 | 0.992 | 0.956 | 1.029 |
| **BMI,** *kg/m^2^* | **0.04** | **1.17** | **1.008** | **1.358** |
| **ALT,** *IU/L* | 0.693 | 0.992 | 0.952 | 1.033 |
| **AST,** *IU/L* | **0.022** | **1.071** | **1.01** | **1.135** |
| **Insulin,** *uU/ml* | 0.6 | 0.986 | 0.934 | 1.041 |
| **Glucose,** *mmol/l* | 0.796 | 0.967 | 0.752 | 1.244 |
| **Homa-index*** | 0.442 | 1.048 | 0.93 | 1.181 |
| **HbA1c,** *mmol/mol* | 0.095 | 1.035 | 0.994 | 1.079 |
| **Education rank** | 0.033 | 0.857 | 0.744 | 0.987 |

**Supplementary table 13.** **Predictive factors for the presence of significant liver disease in the whole diabetic population.** The table shows predictive factors for LSM ≥8.1 kPa on multivariate analysis. Education rank is derived from the Index of multiple deprivation.

**Homa-index was calculated only in those not on insulin treatment.*

*Abbreviations: OR: odds ratio, 95%CI: 95% confidence interval, BMI: Body Mass Index, ALT: Alanine aminotransferase, AST: aspartate aminotransferase, HbA1c: glycated haemoglobin*

|  | **Derivation cohort**  **N=287** | **Validation cohort**  **Royal Free**  **N=218** | **Validation cohort**  **Sicily**  **N=168** |  |  |
| --- | --- | --- | --- | --- | --- |
|  | **Median (IQR)** | **Median (IQR)** | **Median (IQR)** | **P value*** | **P value**** |
| **Age,** *years* | 61 (54-66) | 61 (53-68) | 56 (50-63) | 0.58 | **0.0001** |
| **Waist circum,** *cm* | 106 (98-116) | 111 (100-120) | 109 (100-118) | **0.006** | 0.09 |
| **BMI,** *kg/m^2^* | 30.4 (26.9-34.4) | 32.4 (28.1-37.7) | 31.8 (28.9-35.5) | **0.0001** | **0.004** |
| **PLT,** *x 10^9^/uL* | 243 (202-290) | 230 (181-276) | 229 (178-267) | **0.001** | **0.01** |
| **ALT,** *IU/L* | 30 (22-45) | 49 (33-68) | 56 (37-82) | **0.0001** | **0.0001** |
| **AST,** *IU/L* | 27 (22-35) | 34 (24-48) | 38 (28-51) | **0.0001** | **0.0001** |
| **LSM,** *kPa* | 5.6 (4.4-7.3) | 7.9 (5.4-14) | 11 (7.8-16.8) | **0.0001** | **0.0001** |

**Supplementary table 14. Differences between the derivation cohort and the validation cohorts.** The table shows the differences between the primary care cohort (derivation cohort) and cohorts from Royal Free Hospital and Sicily. Variables are expressed as median and IQR.

* p-value for difference between derivation cohort and validation cohort from Royal Free.

** p-value for difference between derivation cohort and validation cohort from Sicily.

**FIGURES**
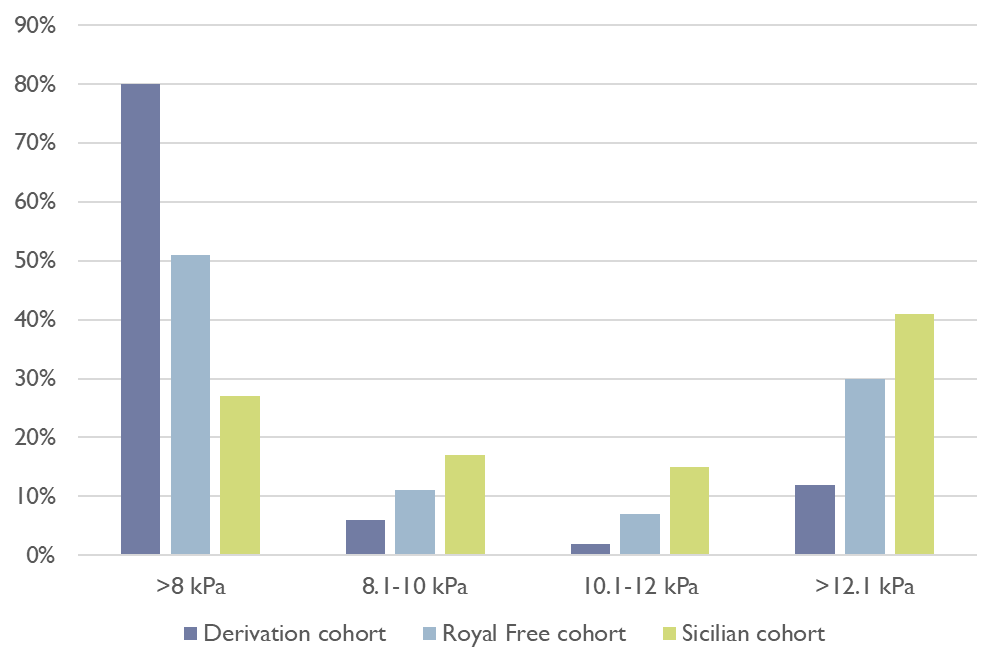


**Supplementary figure 1. Study population versus external validation cohorts stratified per LSM ranges.**

The bar chart illustrates the prevalence of patients with different ranges of LSM in the three groups: derivation cohort, Royal free cohort and Sicilian cohort.

**REFERENCES**

1. Angulo, P., et al., *The NAFLD fibrosis score: a noninvasive system that identifies liver fibrosis in patients with NAFLD.* Hepatology, 2007. **45**(4): p. 846-54.

2. Vallet-Pichard, A., et al., *FIB-4: an inexpensive and accurate marker of fibrosis in HCV infection. comparison with liver biopsy and fibrotest.* Hepatology, 2007. **46**(1): p. 32-6.

3. Boursier, J., et al., *Determination of reliability criteria for liver stiffness evaluation by transient elastography.* Hepatology, 2013. **57**(3): p. 1182-91.

4. Hernaez, R., et al., *Diagnostic accuracy and reliability of ultrasonography for the detection of fatty liver: a meta-analysis.* Hepatology, 2011. **54**(3): p. 1082-1090.

5. guidelines, N., *NICE guidelines: Non-alcoholic fatty liver disease (NAFLD): assessment and management.* 2016.

6. European Association for the Study of the Liver. Electronic address, e.e.e., et al., *EASL Clinical Practice Guidelines on non-invasive tests for evaluation of liver disease severity and prognosis - 2021 update.* J Hepatol, 2021. **75**(3): p. 659-689.

7. Papatheodoridi, M., et al., *Refining the Baveno VI elastography criteria for the definition of compensated advanced chronic liver disease.* J Hepatol, 2021. **74**(5): p. 1109-1116.
