# Supplementary material for: Derivation and validation of the BIMAST score for predicting the presence of fibrosis due to Metabolic dysfunction-associated steatotic liver disease among diabetic patients in the community
Source: PLoS One. 2024 Sep 27;19(9):e0307500. doi: 10.1371/journal.pone.0307500 (PMC11432895; doi:10.1371/journal.pone.0307500)
Supplement: Attachment — Submitted filename: renamed_7adce.pdf [file pone.0307500.s002.pdf]

Check for updates

Received: 30 June 2023 | Revised: 18 August 2023 | Accepted: 31 August 2023

DOI: 10.1111/liv.15730

ORIGINAL ARTICLE

Liver INTERNATIONAL WILEY

A prospective study on the prevalence of MASLD in people with type-2 diabetes in the community. Cost effectiveness of screening strategies

Roberta Forlano[1] | Tijana Stanic[2] | Sahan Jayawardana[2] | Benjamin Harvey Mullish[1] | Michael Yee[3] | Elias Mossialos[2,4] | Robert Goldin[5] | Salvatore Petta[6] | Emmanouil Tsochatzis[7] | Mark Thursz[1] | Pinelopi Manousou[1]

[1]Liver Unit/Division of Digestive Diseases, Department of Metabolism, Digestion and Reproduction, Faculty of Medicine, Imperial College London, London, UK

[2]Department of Health Policy, London School of Economics and Political Science, London, UK

[3]Section of Endocrinology and Metabolic Medicine, St Mary's Hospital, Imperial College NHS Trust, London, UK

[4]Centre for Health Policy, The Institute of Global Health Innovation, Imperial College London, London, UK

[5]Department of Cellular Pathology, Faculty of Medicine, Imperial College London, London, UK

[6]Section of Gastroenterology and Hepatology, PROMISE, University of Palermo, Palermo, Italy

[7]Institute for Liver and Digestive Health, University College London, London, UK

Correspondence
Pinelopi Manousou, Liver Unit/Division of Digestive Diseases, Department of Metabolism, Digestion and Reproduction, St Mary's Hospital Campus, Imperial College London 10 Floor, QEQM Wing, South Wharf Road, Paddington, London W2 1NY, UK.
Email: p.manousou@imperial.ac.uk

Funding information
European Association for the Study of the Liver; NIHR Imperial Biomedical Research Centre

Abstract

Background and Aims: As screening for the liver disease and risk-stratification pathways are not established in patients with type-2 diabetes mellitus (T2DM), we evaluated the diagnostic performance and the cost-utility of different screening strategies for MASLD in the community.

Methods: Consecutive patients with T2DM from primary care underwent screening for liver diseases, ultrasound, ELF score and transient elastography (TE). Five strategies were compared to the standard of care: ultrasound plus abnormal liver function tests (LFTs), Fibrosis score-4 (FIB-4), NAFLD fibrosis score, Enhanced liver fibrosis test (ELF) and TE. Standard of care was defined as abnormal LFTs prompting referral to hospital. A Markov model was built based on the fibrosis stage, defined by TE. We generated the cost per quality-adjusted life year (QALY) gained and calculated the incremental cost-effectiveness ratio (ICER) over a lifetime horizon.

Results: Of 300 patients, 287 were included: 64% (186) had MASLD and 10% (28) had other causes of liver disease. Patients with significant fibrosis, advanced fibrosis, and cirrhosis due to MASLD were 17% (50/287), 11% (31/287) and 3% (8/287), respectively. Among those with significant fibrosis classified by LSM≥8.1 kPa, false negatives were 54% from ELF and 38% from FIB-4. On multivariate analysis, waist circumference, BMI, AST levels and education rank were independent predictors of significant and advanced fibrosis. All the screening strategies were associated with QALY gains, with TE (148.73 years) having the most substantial gains, followed by FIB-4 (134.07 years), ELF (131.68 years) and NAFLD fibrosis score (121.25 years). In

Abbreviations: ALT, alanine aminotransferase; AST, aspartate aminotransferase; BMI, body mass index; CAP, controlled attenuation parameter; CC, compensated cirrhosis; CET, cost-effectiveness threshold; DC, decompensated cirrhosis; ELF, enhanced liver fibrosis; FIB-4, fibrosis score-4; GGT, gamma-glutamil transferase; GP, general practitioner; HCC, hepatocellular carcinoma; ICER, incremental cost-effectiveness ratio; LFTs, liver function tests; LSM, liver stiffness measurement; LT, liver transplantation; MASLD, Metabolic-dysfunction associated steatotic liver disease; MetALD, MASLD with increased alcohol intake; MLD, mild or no liver disease; NNT, number needed to treat/screen; OR, odd ratio; QALY, quality-adjusted life year; SLD, significant or advanced liver disease; T2DM, type-2 diabetes mellitus; TE, transient elastography; US, ultrasound.

This is an open access article under the terms of the Creative Commons Attribution License, which permits use, distribution and reproduction in any medium, provided the original work is properly cited.
© 2023 The Authors. *Liver International* published by John Wiley & Sons Ltd.

Handling Editor: Luca Valenti

the cost-utility analysis, ICER was £2480/QALY for TE, £2541.24/QALY for ELF and £2059.98/QALY for FIB-4.

Conclusion: Screening for MASLD in the diabetic population in primary care is cost-effective and should become part of a holistic assessment. However, traditional screening strategies, including FIB-4 and ELF, underestimate the presence of significant liver disease in this setting.

KEYWORDS
liver fibrosis, metabolic dysfunction-associated steatotic liver disease, primary care, screening

1 | INTRODUCTION

Metabolic dysfunction-associated steatotic liver disease (MASLD) is the most common cause of abnormal liver function tests (LFTs) worldwide, with a global estimated prevalence of 30%.[1] MASLD is also expected to become the leading cause of end-stage liver disease in the coming decades.[2] Histologically, MASLD encompasses a spectrum of disorders from steatosis with or without hepatocellular injury and/or inflammation (Metabolic dysfunction-associated Steato-Hepatitis, MASH) and a variable degree of fibrosis through to cirrhosis.[3] Fibrosis stage represents the strongest predictor of clinical outcomes—liver and non-liver-related—in these patients.[4] From a clinical perspective, the presence of type 2 diabetes mellitus (T2DM), is an independent predictor of advanced fibrosis in patients with MASLD,[5] with a greater prevalence of advanced disease in diabetic compared to non-diabetic individuals, especially in younger ages.[6]

Given the high prevalence—estimated at 55.5%[7]—and severity of MASLD in the diabetic population, there is a major interest in early detection of the disease, especially in primary care,[8] where diagnosing MASLD is perceived as a clinical challenge, with specific concerns on performing risk-stratification among patients.[9] Both the AASLD[10] and the EASL guidelines[11] recommend screening for MASLD in high-risk risk groups (i.e., patients with metabolic syndrome) following a 2-tier system. Specifically, patients should be stratified using non-invasive markers of fibrosis such as Fibrosis score-4 (FIB-4) and/or NAFLD fibrosis score in primary care, followed by Enhanced liver fibrosis test (ELF) and/or transient elastography (TE) in a specialist setting. Such strategy relies heavily on scores, which were derived from a tertiary care setting, and whose diagnostic accuracy in primary care is still unclear.[12] Furthermore, there is still a debate on whether screening might be cost-effective in this population.[8] Finally, despite the most recent diabetes management guidelines suggesting to screen diabetics for MASLD,[13] the overall awareness among diabetologists remains low.[14]

In this study, we aimed to establish the prevalence of MASLD in patients with T2DM in primary care. Moreover, we tested the performance of non-invasive markers and further developed a risk-stratification pathway. We also built a Markov model simulating MASLD screening and assessed the cost-utility of different screening strategies for MASLD in the diabetic community.

Lay summary

Metabolic dysfunction-associated steatotic liver disease (MASLD), a common disease where excessive fat accumulated in the liver and may result in cirrhosis and heart attacks, is a highly prevalent, yet largely underappreciated liver condition that is closely associated with metabolic disease and type 2 diabetes mellitus (T2DM). Yet, a strategy to understand who is at risk of developing this disease and suffering liver damage is lacking.

In this study, we describe the prevalence of advanced liver disease in diabetics in primary care and we define an easy way to screen diabetics for MASLD. We demonstrate that, among diabetics, education level is associated with a greater risk of having liver disease. Moreover, we demonstrate that screening for fatty liver in primary care using non-invasive markers of fibrosis, is cost-effective and should be offered to all the diabetics in the community.

2 | MATERIALS AND METHODS

2.1 | Study population

This single-centre, cross-sectional study prospectively recruited consecutive patients with T2DM in primary care and community clinics from the North-West London general practitioner (GP) network. Inclusion criteria were the ability to give informed consent, age >18 years and presence of T2DM, as defined by medical history or recent 2 h post-challenge plasma glucose ≥11.1 mmol/L. Patients were excluded if they had known the liver disease.

2.2 | Screening for liver disease and MASLD

All the patients were screened for liver disease and the presence of MASLD with blood tests (full liver screen), imaging ultrasound (US) and TE, with liver stiffness measurement (LSM) and Controlled

14783231, 2024, 1, Downloaded from https://onlinelibrary.wiley.com/doi/10.1111/liv.15730 by Cochrane Saudi Arabia, Wiley Online Library on [20/05/2024]. See the Terms and Conditions (https://onlinelibrary.wiley.com/terms-and-conditions) on Wiley Online Library for rules of use; OA articles are governed by the applicable Creative Commons License

Liver INTERNATIONAL -WILEY
14783231, 2024, 1, Downloaded from https://onlinelibrary.wiley.com/doi/10.1111/liv.15730 by Cochrane Saudi Arabia, Wiley Online Library on [20/05/2024]. See the Terms and Conditions (https://onlinelibrary.wiley.com/terms-and-conditions) on Wiley Online Library for rules of use; OA articles are governed by the applicable Creative Commons License

attenuation parameter (CAP) score. Moderate-to-high cardiovascular risk was defined based on Qrisk2 score as ≥20%. Further details on screening procedures are given in Supplementary material.

2.3 | Cost-effectiveness analysis

2.3.1 | Screening strategies and identification rates

In this study, five screening strategies were compared against the standard of care: (1) US plus LFTs, (2) FIB-4, (3) NAFLD fibrosis score, (4) ELF and (5) TE. Standard of care was derived from previously published economic evaluations of MASLD screening where this entailed LFTs or no screening[15,16] (Supplementary Table S1). As part of the standard of care, abnormal LFTs were assumed to prompt referral to the hospital, with a 65% specificity and 35% sensitivity for liver fibrosis.[17]

In the first tier of each strategy, patients were divided into two groups: no disease/MASLD without significant fibrosis versus MASLD with significant and advanced fibrosis. No disease and MASLD without significant fibrosis were considered the same group for this analysis as the management would be similar and would not trigger a referral to secondary care compared to MASLD with significant and advanced fibrosis that triggers a referral to specialist care.[11] In strategy 1 (US plus LFTs), MASLD with significant fibrosis was defined as evidence of steatosis and features of chronic liver disease on ultrasound, plus elevated LFTs. In strategy 2, significant fibrosis was defined as FIB-4 >1.3 and in strategy 3, as NAFLD fibrosis score >−1.45. In strategy 4 (ELF) significant fibrosis was defined as ELF≥9.8 and in strategy 5 (TE), as LSM ≥8.1 kPa.[11]

2.3.2 | Decision-analytic model

We developed a decision tree to characterise the risk stratification and diagnostic performance of each of the primary care screening strategies evaluated. We adapted previously published Markov models[15,17] to characterise the subsequent health states and disease pathways of patients based on their initial primary care screening risk stratification (Figure 1).

The model was built upon four health states:

1. Mild or no liver disease (MLD) if MASLD with LSM≤8 kPa or there was no disease;
2. Significant and advanced liver disease (SLD) if LSM≥8.1 kPa;
3. Compensated cirrhosis (CC) (histological (where available) or biochemical/radiological evidence of cirrhosis without evidence of decompensation);
4. False negatives if LSM ≥8.1 kPa but were within the normal range at screening with other non-invasive markers of fibrosis.

Patients with advanced stages of liver disease could progress to health states that reflect end-stage liver disease, including

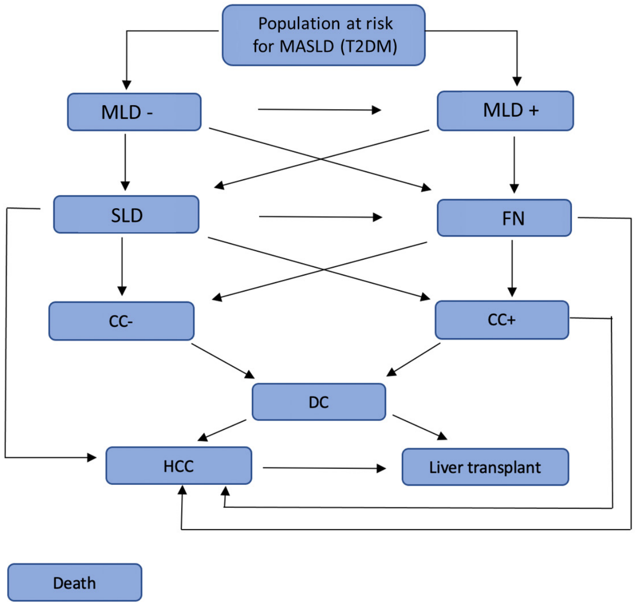

FIGURE 1 Markov model for the cost-effectiveness analysis. CC, compensated cirrhosis (clinical diagnosis of cirrhosis); DC, decompensated cirrhosis; FN, false negatives (MASLD with LSM ≥8.1 kPa who were false negatives at screening); HCC, hepatocellular carcinoma; MLD, mild liver disease (no MASLD or MASLD with LSM≤8 kPa); MASLD, metabolic-dysfunction associated steatotic liver disease; SLD, significant liver disease (MASLD with LSM ≥8.1 kPa who were true positives at screening); T2DM, type 2 diabetes mellitus; +, diagnosed; −, undiagnosed.

decompensated cirrhosis (DC), hepatocellular carcinoma (HCC), liver transplant (LT) and death.[15] With a diagnosis present (status of significant liver disease and/or compensated cirrhosis), there is a probability that the management of the patient is modified to reduce the risk of progression to CC, decompensation or death. In this case, progression rates of SLD and CC groups were assumed as slower, compared to those whose diagnosis was missed at screening (false negatives).[15] Details on model input parameters are given in Supplementary material.

2.3.3 | Model outcomes

The cost-utility analysis in the base-case was conducted over a lifetime horizon and generated the cost per quality-adjusted life year (QALY) gained. A discount rate of 3.5% per year was applied to outcomes and costs, as recommended by the NICE guidelines.[18] We calculated the average cost-effectiveness and the incremental cost-effectiveness ratio (ICER) compared to the standard of care.[15,17] Life expectancy, lifetime costs and the number of correct diagnoses were also estimated. According to NICE guidelines, a cost-effectiveness threshold (CET) of £20 000/QALY gained was set for the base-case analysis as per previous studies.[19] Key input parameters with the highest level of uncertainty (i.e., transition

probabilities, utility values, costs and screening ratios) were varied to determine the impact of their variability on cost-effectiveness results. Sensitivity analysis ranges and probabilistic distributions were derived from previous literature and are reported in detail in Supplementary Tables S4–S8.

3 | RESULTS

3.1 | Study population

Between April 2019 and January 2021, a total of 300 consecutive patients with T2DM were enrolled from the North-West London GP network. Overall, 287 patients underwent the whole screening procedure, while 13 did not complete the screening and were excluded (Supplementary Figure S1). The study population was diverse in terms of ethnic background and also diverse in terms of severity of T2DM and anti-diabetic treatments (Tables 1 and 2). The success rate for performing TE in this population was 99% (286/287).

3.2 | Prevalence of MASLD and fibrosis

The overall prevalence of MASLD, based on US, was 64% (186/287), while the prevalence of other liver diseases was 9% (28/287: 27 with MASLD and increased alcohol intake, MetALD and 1 with chronic hepatitis B). There were no cases of secondary MASLD. The overall prevalence of significant liver disease (LSM ≥8.1 kPa), was 17% (50/287), while the prevalence of advanced fibrosis (LSM ≥12.1 kPa), was 10% (31/287) in the whole population. Those with significant liver disease had higher alanine aminotransferase (ALT, 46 vs. 30 IU/L, $p=.0001$), aspartate aminotransferase (AST, 37 vs. 26 IU/L, $p=.0001$) and gamma-GGT values (GGT, 62 vs. 27 IU/L, $p=.0001$). Of note, 42% of the patients with 8.1 kPa ≤LSM ≤12.1 kPa and 38% of patients with LSM ≥12.1 kPa had normal LFTs.

When the CAP score was used to define steatosis, the overall prevalence of MASLD was 67% (195/287), the prevalence of significant liver disease (LSM ≥8.1 kPa), was 16% (48/287), while the prevalence of advanced fibrosis (LSM ≥12.1 kPa), was 11% (33/287) in the whole population. Of note, 3% (9/287) had elevated CAP score but no evidence of steatosis on the US. However, only those with a positive ultrasound were considered as having steatosis.

3.3 | Prevalence of cirrhosis

The prevalence of newly diagnosed cirrhosis secondary to MASLD was 3% (8/287; 6 with clinical diagnosis and 2 based on histology) in the whole diabetic population and 5% (8/184) in the MASLD subgroup (Supplementary Figure S1). The number needed to treat/screen (NNT) in this population was 4.56 (3.38–7). Due to the COVID-19-related restrictions, only 11 patients underwent a liver biopsy among those with elevated LSM (as per standard of care): all the biopsied cases had liver fibrosis stage ≥2 according to the CRN scoring system.

3.4 | Obesity and glycaemic control in MASLD with significant and advanced fibrosis

When compared to those with MASLD and normal LSM ($n=136$), patients with significant fibrosis (LSM ≥8.1 kPa) ($n=50$) presented higher body mass index (BMI) (36.8 vs. 30.3 kg/m^2, $p=.0001$), larger hip (123 vs. 110 cm, $p=.0001$) and waist circumferences (120 vs. 105 cm, $p=.0001$). In terms of metabolic control, patients with MASLD and significant fibrosis showed higher median HbA1c (71 vs. 59 mmol/mol, $p=.0001$), fasting glucose (9.4 vs. 6.7 mmol/L, $p=.001$), insulin level (21 vs. 12.4 μU/mL, $p=.001$) and HOMA index (8.1 vs. 3.3, $p=.001$). There was no difference in terms of duration of diabetes, anti-diabetic medications or presence of diabetic complications (Table 2). Similar results were observed when comparing those with advanced fibrosis (LSM ≥12.1 kPa) ($n=31$) compared to those without.

Overall, 29 patients (10%) had a historical cardiovascular event, while 134 (46%) had moderate to high cardiovascular risk (Qrisk2 score ≥10%). The AUROC for predicting the presence of moderate to high cardiovascular risk was 0.58 (95%CI: 0.49–0.66, $p=.05$) for ELF, 0.53 (95%CI: 0.44–0.61, $p=.053$) for FIB-4, 0.53 (95%CI: 0.45–0.62. $p=.06$) for NAFLD fibrosis score and 0.54 (95%CI: 0.45–0.62, $p=.07$) for LSM.

3.5 | Advanced liver disease is more prevalent in the deprived population

In terms of socio-economic status, those with MASLD and significant fibrosis lived in more deprived neighbourhoods according to their median education rank (18 789 vs. 23 148, $p=.03$) (Supplementary Table S9). Similarly, those with MASLD and advanced fibrosis lived in more deprived neighbourhoods according to their median education rank (18 793 vs. 23 162, $p=.05$). Conversely, there was no difference in terms of the other deprivation scores: income, employment, health deprivation and disability, barriers to housing and service, and crime.

On multivariate analysis, waist circumference (crude OR 1.086, 95%CI 1.021–1.154, $p=.008$), BMI (crude OR 1.17, 95% CI 1.008–1.358, $p=.04$), AST (crude OR 1.071, 95%CI 1.07–1.01, $p=.022$) and education rank (crude OR 0.857, 95%CI 0.744–0.987) were independent predictors of significant liver disease in the whole diabetic population (Table 3).

Similarly, waist circumference (OR 1.09, 95%CI 1.04–1.14, $p=.0001$), BMI (OR 1.07, 95%CI 1.002–1.2, $p=.05$), AST (OR 1.025, 95%CI 1.01–1.051, $p=.002$) and education rank (OR 0.92, 95%CI 0.82–0.99) were independent predictors of advanced liver disease in the whole diabetic population.

14783231, 2024, 1, Downloaded from https://onlinelibrary.wiley.com/doi/10.1111/liv.15730 by Cochrane Saudi Arabia, Wiley Online Library on [20/05/2024]. See the Terms and Conditions (https://onlinelibrary.wiley.com/terms-and-conditions) on Wiley Online Library for rules of use; OA articles are governed by the applicable Creative Commons License

Liver INTERNATIONAL —WILEY

TABLE 1 Characteristics of the study population and differences between patients with and without MASLD.

	Study population N = 287	MASLD N = 186	Normal liver N = 73	
	Median (IQR)	Median (IQR)	Median (IQR)	*p*-value*
Age, years	59 (59–66)	60 (54–66)	59 (53–65)	.83
Waist circum, cm	107 (107–116)	108 (101–118)	98 (92–106)	.0001
Hip circum, cm	110 (102–119)	112 (105–122)	103 (98–108)	.0001
BMI, kg/m^2	30.8 (26.9–34.4)	31.4 (28.4–35.8)	26.9 (24.8–30.3)	.0001
PLT, $\times 10^9/\mu L$	250 (202–290)	245 (212–287)	249 (206–298)	.88
ALT, IU/L	35 (22–45)	34 (23–49)	24 (18–28)	.0001
AST, IU/L	31 (22–35)	28 (23–37)	24 (19–27)	.0001
GGT, IU/L	47 (19–50)	32 (22–52)	19 (17–27)	.0001
ALP, IU/L	88 (70–103)	84 (72–105)	85 (63–99)	.7
Albumin, g/L	40 (39–42)	41 (39–42)	40 (39–42)	.83
Bilirubin, µmol/L	10.6 (6–12)	9 (6–12)	8 (6–14)	.55
Total Cholesterol, mmol/l	4.1 (3.5–4.7)	4.1 (3.4–4.7)	4 (3.6–4.5)	.58
TRG, mmol/L	2.3 (1.02–2.08)	1.4 (1.07–2.1)	1.2 (0.98–1.5)	.25
HDL, mmol/L	1.1 (0.9–1.3)	1.1 (0.9–1.2)	1.16 (1.06–1.39)	.25
LDL, mmol/L	2.3 (1.6–2.7)	2.2 (1.6–2.8)	2.1 (1.7–2.6)	.68
Ferritin, ng/mL	124 (43–155)	82 (39–140)	70 (28–178)	.91
**Diabetes characteristics**				
	**Median (IQR)**	**Median (IQR)**	**Median (IQR)**	***p*-value***
Fasting glucose, mmol/L	7.9 (5.5)	7.4 (5.6–10.2)	6.2 (4.8–7.8)	.001
HbA1c, mmol/mol	60 (49–70)	60 (50–74)	55 (48–61)	.0001
Insulin, µU/mL	24 (8.1–26.5)	15.3 (9.8–28.2)	7.2 (5.8–12.2)	.028
Homa index	8 (1.9–8.95)	4.6 (2.2–10.3)	2.1 (1.35–4.8)	.0001
Duration DM, years	11 (4–16)	10 (3–16)	13 (7–16)	.16
	N (%)	*N* (%)	*N* (%)	*p*-value*
Diet controlled	39 (13)	25 (13)	13 (18)	.11
On oral agents	227 (79)	170 (91)	55 (75)	.16
On GLP-1RA	37 (13)	31 (16)	6 (8)	.08
On insulin	74 (25)	51 (28)	23 (31)	.18
Diabetic complications	45 (16)	26 (14)	15 (21)	.82
**Ethnic background and comorbidities**				
	***N* (%)**	***N* (%)**	***N* (%)**	***p*-value***
Male gender	160 (53)	104 (56)	34 (45)	.07
White, Caucasian	102 (32)	64 (34)	15 (20)	.02
White, Hispanic	6 (2)	3 (1)	2 (2)	.43
Black African, Afro-Caribbean	33 (12)	22 (12)	10 (13)	.41
Arab	74 (28)	52 (28)	20 (26)	.52
South Asian	47 (17)	31 (17)	16 (21)	.2
East Asian	24 (8)	14 (7)	10 (13)	.09
Hypertension	191 (67)	120 (64)	50 (66)	.32
Dyslipidaemia	148 (52)	98 (53)	39 (52)	.51
Psychiatric disorder	41 (15)	27 (14)	11 (14)	.53
Previous ACE	28 (10)	16 (8)	11 (14)	.98
On statin	214 (75)	138 (74)	57 (76)	.31

Note: The table shows the differences between patients with (*n* = 186) and without (*n* = 73) MASLD in the whole study population (*n* = 287). Variables are expressed as median and IQR or relative percentages.

Abbreviations: ACE, acute cardiovascular event; ALP, alkaline phosphatase; ALT, alanine aminotransferase; AST, aspartate aminotransferase; BMI, body mass index; GGT, gamma-glutamyl transferase; GLP-1RA, glucagon like peptide-1 receptor agonist; HbA1c, glycated haemoglobin; HDL, high density lipoprotein; IQR, interquartile range, LDL, low density lipoprotein; PLT, platelet; TRG, triglycerides.

**p*-value refers to differences between patients with MASLD and normal liver.

14783231, 2024, 1, Downloaded from https://onlinelibrary.wiley.com/doi/10.1111/liv.15730 by Cochrane Saudi Arabia, Wiley Online Library on [20/05/2024]. See the Terms and Conditions (https://onlinelibrary.wiley.com/terms-and-conditions) on Wiley Online Library for rules of use; OA articles are governed by the applicable Creative Commons License

TABLE 2 Differences between patients with MASLD stratified per liver stiffness measurement greater than 8.1 kPa.

	MASLD, LSM≥8.1 kPa *N* = 50	MASLD, normal LSM *N* = 136	
	**Median (IQR)**	**Median (IQR)**	***p*-value***
Age, years	60 (51–65)	61 (54–65)	.49
Waist circum, cm	120 (112–127)	105 (99–113)	.0001
Hip circum, cm	123 (123–132)	110 (103–119)	.0001
BMI, kg/m^2	36.8 (32–39.7)	30.3 (27.6–33.6)	.0001
PLT, ×10^9/uL	231 (198–266)	255 (215–300)	.3
ALT, IU/L	46 (25–60)	30 (22–43)	.0001
AST, IU/L	37 (28–48)	26 (22–32)	.0001
GGT, IU/L	62 (35–96)	27 (19–39)	.0001
ALP, IU/L	83 (70–110)	84 (72–101)	.62
Albumin, g/L	40 (38–41)	41 (39–42)	.06
Bilirubin, µmol/L	10 (7–16)	8 (6–11)	.55
Total Cholesterol, mmol/L	3.9 (3.4–4.4)	4.1 (3.5–4.8)	.14
TRG, mmol/L	1.3 (1.08–2.2)	1.5 (1.06–2.1)	.92
HDL, mmol/L	1.1 (0.9–1.2)	1.08 (0.9–1.3)	51
LDL, mmol/L	1.9 (1.6–2.6)	2.2 (1.6–2.8)	.42
Ferritin, ng/mL	108 (48–182)	81 (36–124)	.31
**Diabetes characteristics**			
	**Median (IQR)**	**Median (IQR)**	***p*-value***
Fasting glucose, mmol/L	9.4 (6.2–13.4)	6.7 (5.2–9.2)	.001
HbA1c, mmol/mol	71 (56–84)	59 (49–68)	.0001
Insulin, µU/mL	21 (14–37.2)	12.4 (9–25)	.001
Homa index	8.1 (4.5–14.1)	3.3 (2.1–8.4)	.001
Duration DM, years	10 (4–16)	10 (3–16)	.46
	N (%)	*N* (%)	*p*-value*
Diet controlled	1 (2)	24 (17)	.052
On oral agents	43 (86)	127 (93)	.051
On GLP-1-RA	10 (20)	21 (15)	.07
On insulin	15 (30)	36 (26)	.25
Diabetic complications	10 (20)	16 (12)	.82
**Ethnic background and comorbidities**			
	***N* (%)**	***N* (%)**	***p*-value***
Male gender	29 (58)	75 (55)	.44
White, Caucasian	20 (40)	45 (33)	.22
White, Hispanic	1 (2)	2 (1)	.61
Black African, Afro-Caribbean	4 (8)	18 (13)	.23
Arab	15 (30)	37 (27)	.43
South Asian	8 (16)	22 (16)	.47
East Asian	2 (4)	12 (9)	.21
Hypertension	33 (66)	87 (63)	.45
Dyslipidaemia	27 (54)	71 (52)	.46
Psychiatric disorder	9 (18)	19 (13)	.28
Previous ACE	3 (6)	13 (9)	.29
On statin	39 (78)	99 (76)	.32

Note: The table shows the differences between patients with MASLD with elevated ($n = 50$) and normal ($n = 136$) LSM. Variables are expressed as median and IQR or relative percentages.

Abbreviations: ACE, acute cardiovascular event; ALP, alkaline phosphatase; ALT, alanine aminotransferase; AST, aspartate aminotransferase; BMI, body mass index; GGT, gamma-glutamyl transferase; GLP-1RA, glucagon like peptide-1 receptor agonist; HbA1c, glycated haemoglobin; HDL, high density lipoprotein; IQR, interquartile range, LDL, low density lipoprotein; PLT, platelet; TRG, triglycerides.

**p*-value: differences between patients with MASLD with elevated LSM and normal LSM.

14783231, 2024, 1, Downloaded from https://onlinelibrary.wiley.com/doi/10.1111/liv.15730 by Cochrane Saudi Arabia, Wiley Online Library on [20/05/2024]. See the Terms and Conditions (https://onlinelibrary.wiley.com/terms-and-conditions) on Wiley Online Library for rules of use; OA articles are governed by the applicable Creative Commons License

TABLE 3 Predictive factors for the presence of significant liver disease in the whole diabetic population.

Variable	Sig.	Crude OR	95% CI Lower	Upper
Waist circumference, cm	0.008	1.086	1.021	1.154
Hip circumference, cm	0.659	0.992	0.956	1.029
BMI, kg/m^2	0.04	1.17	1.008	1.358
ALT, IU/L	0.693	0.992	0.952	1.033
AST, IU/L	0.022	1.071	1.01	1.135
Insulin, uU/ml	0.6	0.986	0.934	1.041
Glucose, mmol/L	0.796	0.967	0.752	1.244
Homa-index[a]	0.442	1.048	0.93	1.181
HbA1c, mmol/mol	0.095	1.035	0.994	1.079
Education rank	0.033	0.857	0.744	0.987

Note: The table shows predictive factors for LSM ≥8.1 kPa on multivariate analysis. Education rank is derived from the Index of multiple deprivation.

Abbreviations: 95%CI, 95% confidence interval; ALT, Alanine aminotransferase; AST, aspartate aminotransferase; BMI, body mass index; HbA1c, glycated haemoglobin; OR, odds ratio.

[a]Homa-index was calculated only in those not on insulin treatment.

3.6 | Younger patients with normal LFTs are missed by Fib-4 and ELF screening

Overall, 19 (19/50 = 38%) patients with LSM ≥8.1 kPa were missed by FIB-4 (false negatives) and 27 (27/50 = 54%) by ELF. Specifically, those who were misclassified by FIB-4 as low-risk were significantly younger (57 vs. 62 years, $p = .03$) and had lower AST levels (35 vs. 41 IU/L, $p = .034$) compared to those correctly classified as a low-risk group. We could not identify any significant variables between those classified correctly or not correctly by ELF.

Conversely, 36 (36/126 = 28%) with FIB-4 ≥ 1.3 and 32 (32/52 = 62%) patients with ELF≥9.8 had normal LSM.

3.7 | Sub-analysis per gender

In this study, 53% (160/287) were men. When compared to women, the overall prevalence of SLD was significantly higher (78% vs. 67%, $p = .022$). Men had greater waist (108 vs. 104 cm, $p = .001$) but similar BMI compared to women. However, there was no difference in the overall prevalence of significant (LSM ≥8.1 kPa; 18% vs. 17%, $p = .44$), or advanced fibrosis (LSM ≥12.1 kPa; 12% vs. 11%, $p = .6$). Interestingly, despite men having significantly higher ALT levels (34 vs. 26 IU/L, $p = .001$), the rate of false negatives to FIB-4 or other screening strategies among those with significant fibrosis was similar to women (FIB-4 <1.3 if LSM ≥8.1 kPa; 38% vs. 37%, $p = .34$).

When women were stratified according to menopausal status, menopausal women (88/127 = 69%) had significantly lower BMI (30.4 vs. 32.8 kg/m^2, $p = .04$) and higher HDL (1.3 vs. 1.15 mmol/L, $p = .001$) compared to non-menopausal women (19/127 = 31%). There was no difference in terms of the prevalence of MASLD, significant or advanced liver fibrosis.

When compared to age-matched men, menopausal women showed smaller waist (103 vs. 108 cm, $p = .03$) and hip circumference (111 vs. 113 cm, $p = .018$). Menopausal women also showed significantly lower ALT (26 vs. 33 IU/L, $p = .003$) and CAP score (293 vs. 313 dB/m, $p = .013$) compared to age-matched men. There was no difference in terms of the prevalence of MASLD, significant or advanced fibrosis.

3.8 | Cost-effectiveness analysis

The cost-effectiveness analysis was based on the performance characteristics (positive predictive values and negative predictive values) for the identification of patients with significant and advanced fibrosis from the study population (Supplementary Figures S2–S6).

Overall, screening for MASLD by any of the strategies analysed improved the rate of diagnosis by 8%–15%. All screening strategies were associated with QALY gains, ranging from 121 to 149 years, with TE (148.73 years) resulting in the most substantial gains, followed by FIB-4 (134.07 years), ELF (131.68 years) and NAFLD fibrosis score (121.25 years). The ICER of TE compared to the standard of care was £2480 per QALY gained (Table 4).

The ICER was most sensitive to variations in progression rates (effect of early diagnosis on disease progression), screening test sensitivity and specificity and model time horizon. Nevertheless, when transition probabilities, utilities, screening treatment effect and cost inputs were modified, we found a >99% probability of MASLD screening tests being cost-effective compared to standard of care in all evaluated scenarios (Figure 2, Supplementary Tables S4–S8). When sensitivity and specificity of each screening test were varied in a range between 20% and 100%, the ICER remained cost-effective below £3260 in all scenarios (Supplementary Tables S4-S8). Although all screening strategies were found to be cost-effective compared to standard of care in the base-case, when the time horizon was decreased from 40 years (lifetime) to 5 years, only FIB-4 remained cost-effective within the NICE cost-effectiveness threshold criteria.

4 | DISCUSSION

Non-alcoholic fatty liver disease has now become the leading cause of chronic liver disease in Western countries and the fastest-growing indication for liver transplantation in the United States.[20] Defining and implementing models of care has been identified as an area of priority for tackling MASLD worldwide.[21] Specifically, there is need for clearly defined, pragmatical referral management pathways, which are based on clinical context and shared with local primary care providers. Being a high-risk group for advanced liver disease,[6,22]

14783231, 2024, 1, Downloaded from https://onlinelibrary.wiley.com/doi/10.1111/liv.15730 by Cochrane Saudi Arabia, Wiley Online Library on [20/05/2024]. See the Terms and Conditions (https://onlinelibrary.wiley.com/terms-and-conditions) on Wiley Online Library for rules of use; OA articles are governed by the applicable Creative Commons License

TABLE 4 Base-case cost-effectiveness analysis of MASLD screening strategies versus standard of care (baseline screening).

	Screening	Standard of care
Screening strategy 1: US + LFTs		
Discounted life expectancy, entire cohort (years)	3751.88	3594.85
QALYs gained, entire cohort (years)	138.19	–
Increase in correct diagnoses compared to baseline screening (%)	10.73	–
Lifetime discounted per person cost (£)	13 542.93	12295.53
Incremental cost per person (£)	0.0008	–
Incremental cost-effectiveness ratio (£/QALY)	2337.92	–
Screening strategy 2: FIB-4		
Discounted life expectancy, entire cohort (years)	3747.62	3594.85
QALYs gained, entire cohort (years)	134.07	–
Increase in correct diagnoses compared to baseline screening (%)	8.29	–
Lifetime discounted per person cost (£)	13 361.87	12 295.53
Incremental cost per person (£)	0.0009	–
Incremental cost-effectiveness ratio (£/QALY)	2059.98	–
Screening strategy 3: NAFLD fibrosis score		
Discounted life expectancy, entire cohort (years)	3734.50	3594.85
QALYs gained, entire cohort (years)	121.25	–
Increase in correct diagnoses compared to baseline screening (%)	−2.32	–
Lifetime discounted per person cost (£)	13 275.08	12 295.53
Incremental cost per person (£)	0.0010	–
Incremental cost-effectiveness ratio (£/QALY)	2092.47	–
Screening strategy 5: ELF test		
Discounted life expectancy, entire cohort (years)	3745.06	3594.85
QALYs gained, entire cohort (years)	131.68	–
Increase in correct diagnoses compared to baseline screening (%)	8.48	–
Lifetime discounted per person cost (£)	13 587.54	12 295.53
Incremental cost per person (£)	0.0008	–
Incremental cost-effectiveness ratio (£/QALY)	2541.24	–
Screening strategy 6: Transient elastography		
Discounted life expectancy, entire cohort (years)	3762.89	3594.85
QALYs gained, entire cohort (years)	148.73	–
Increase in correct diagnoses compared to baseline screening (%)	15.05	–
Lifetime discounted per person cost (£)	13 717.67	12 295.53
Incremental cost per person (£)	0.0007	–
Incremental cost-effectiveness ratio (£/QALY)	2476.57	–

Abbreviations: ELF test, enhanced liver fibrosis test; LFTs, liver function tests; US, ultrasound.

patients with T2DM represent an ideal target for MASLD screening in primary care.

In this study, we studied a cohort of patients with diabetes who were screened for MASLD and other liver diseases in primary care, without any a priori selection. This cohort includes patients with a wide range of antidiabetic treatments, comorbidities, ranges of glycaemic control and length of disease. Furthermore, conducting this study in North-West London, provided us with a very diverse in terms of ethnic and social background, which is a bonus compared to other studies in the field. Overall, the prevalence of MASLD based on the US was 64%, while the prevalence of significant liver disease was 17%, advanced liver disease 11% and cirrhosis 3% in the whole cohort. In a recently published work, in diabetic patients over 50 years old in the community and endocrinology clinics, their results are similar to our cohort with the prevalence of MASLD, advanced fibrosis and cirrhosis at 65%, 14% and 6%.[23]

In our cohort, visceral obesity, education attainment and AST were the main clinical predictors for the presence of significant and advanced fibrosis in primary care. Overall, despite education level being a well-known risk factor for other chronic liver diseases,[24] this is the first work demonstrating clearly that education level is an important determinant of liver disease in the general diabetic

14783231, 2024, 1, Downloaded from https://onlinelibrary.wiley.com/doi/10.1111/liv.15730 by Cochrane Saudi Arabia, Wiley Online Library on [20/05/2024]. See the Terms and Conditions (https://onlinelibrary.wiley.com/terms-and-conditions) on Wiley Online Library for rules of use; OA articles are governed by the applicable Creative Commons License

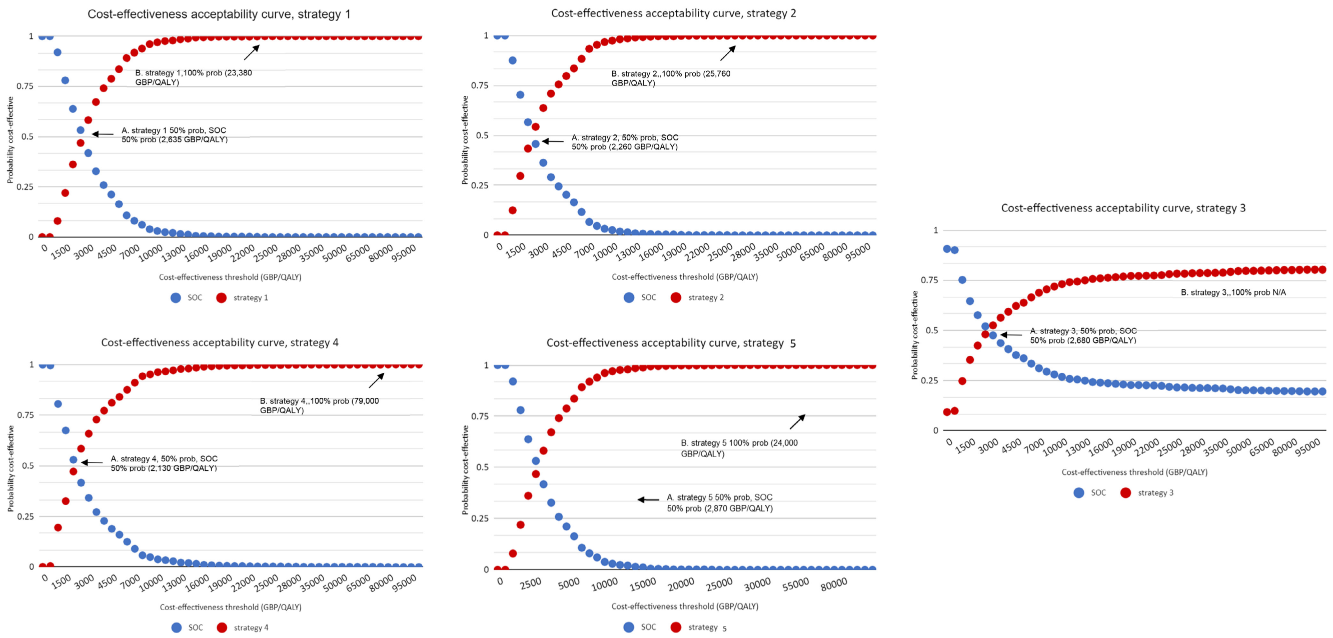

FIGURE 2 Cost-effectiveness acceptability curve. Red and blue lines represent the cost-effectiveness acceptability of MASLD screening strategies 1–5 and standard of care, respectively. Streategy 1 ultrasound plus liver function tests; strategy 2 FIB-4, strategy 3 NAFLD fibrosis score; strategy 4 ELF; strategy 5: Transient elastrography. Each dot on the graph shows the probability of each of the strategies (1–5) being cost-effective (*Y*-axis) at a given cost-effectiveness threshold (*X*-axis). (1) Point A shows the point at which both strategies have 50% probability of being cost-effective; (2) point B shows the point at which scenario 1 has 100% probability of being cost-effective.

population. Clinicians managing patients with T2DM should be aware of the risks associated with poor education and incorporate this knowledge into their patient clinical management. Multidisciplinary teams should ensure that families with poor literacy have an adequate understanding of them being at higher risk for liver disease.

According to the latest published EASL guidelines, patients with T2DM should be screened for MASLD using a two-tier system, that is, FIB-4 and/or ELF in primary care, followed by TE in a specialist setting.[11] Nevertheless, standard of care for diagnosing MASLD among GPs still relies on ultrasound and LFTs, possibly due to limited awareness on the disease and/or screening policies.[25,26] In this cohort, despite AST being a predictive factor against the liver disease, up to 42% of the patients with 8.1 kPa ≤LSM≤12.1 kPa and 38% of the patients with LSM ≥12.1 kPa had normal LFTs at screening. Risk stratification should not rely on LFTs, as they both under- and over-estimate the severity of liver disease in MASLD.[26] Moreover, according to the results from this study, applying FIB-4 with a cut-off of 1.3 in this population would miss up to 38% of the patients with significant liver disease and these would mainly be younger patients with normal LFTs. These results are in line with recently published data and highlight the limitation of the use of FIB-4 in primary care.[27] Similarly, when applying a cut-off of ELF ≥9.8, up to 59% of those with significant liver disease would be missed at screening. Despite recent literature highlighting gender-related differences in MASLD phenotypes,[28] in this population, there was no difference in terms of false negative rates between men and women in this population. Of note, recent evidence has raised the concern that currently used non-invasive markers may underestimate liver disease in diabetics and that more evidence in primary care is needed.[10,29] Nevertheless, it is worth noting that when FIB-4 and ELF were used as standards, LSM underestimated the presence of significant fibrosis in 28% and 62% of the patients, respectively.

Though cost-effectiveness data in screening for MASLD in patients with T2DM in the community is emerging,[8] there is still a debate about the appropriate screening strategy. It is of great importance to identify patients with a high risk of progressive disease, as this would lead to a reduction in progression rates to end-stage liver disease and associated healthcare burden. Moreover, not only lifestyle intervention could delay or reverse fibrosis progression[30,31] but also pharmacotherapies will soon be available. Furthermore, as more severe forms of SLD are also associated with the greatest additional risk of cardiovascular events,[32] screening tools which identify advanced SLD may by proxy also identify those at higher risk for acute cardiovascular events, further extending the utility of screening within this scenario.

In this study, we present a cost-effectiveness analysis for screening for MASLD based on a real-life population of patients with T2DM in primary care. MASLD screening in people with T2DM improved diagnostic outcomes and was cost-effective in all evaluated scenarios under a CET at £20 000. Overall, TE was the screening strategy associated with the greatest clinical gains (148.73 QALYs). These results are in line with published work[27] and emphasise that screening for MASLD is cost-effective compared to standard of care defined as abnormal LFTs or even the combination of US and LFTs.[15,17,33,34] Nevertheless, previous

14783231, 2024, 1, Downloaded from https://onlinelibrary.wiley.com/doi/10.1111/liv.15730 by Cochrane Saudi Arabia, Wiley Online Library on [20/05/2024]. See the Terms and Conditions (https://onlinelibrary.wiley.com/terms-and-conditions) on Wiley Online Library for rules of use; OA articles are governed by the applicable Creative Commons License

studies were based on either hypothetical cohorts or tertiary care populations, while this cost-effectiveness analysis relies on a real-life population from primary care.

This study has several limitations. Firstly, we used TE as the gold standard as only 11 patients were biopsied. However, several other studies as well as the most recent EASL and AASLD guidelines support the use of elastographic techniques in populations with low-disease prevalence.[35,36] Moreover, liver biopsy is not feasible in the screening setting and is unethical to use in a prevalence study.[23] Nevertheless, the use of liver stiffness measurement as a gold standard for the economic model might have led to an overestimation of the cost-effectiveness of the TE in this study. Secondly, the cost-effectiveness analysis also was subject to some limitations, i.e., the need to derive transition probabilities and utility values from previous literature. After an extensive review of the literature, we did not identify any data for diabetic patients at risk for MASLD with the level of granularity required for this cost-effectiveness analysis. To minimise the impact of such data uncertainties, all model input parameters were varied widely both in deterministic and in probabilistic sensitivity analyses.

To summarise, in this study, we found that liver disease due to MASLD is highly prevalent among patients with diabetes in primary care. Our results demonstrate that both FIB-4 and ELF underestimate a substantial subgroup of diabetics with significant fibrosis in the community. We also provide evidence that screening for MASLD is cost-effective, with non-invasive markers of fibrosis being the most cost-effective approach compared to US and LFTs or standard of care. Screening for MASLD-induced fibrosis should become part of the holistic assessment of patients with type-2 diabetes in primary care.

AUTHOR CONTRIBUTIONS

Roberta Forlano: Data curation, formal analysis, funding acquisition, investigation, methodology, project administration, resources, software, visualization, writing—original draft. Tijana Stanic: Formal analysis, methodology, software, visualization, writing - original draft. Sahan Jayawardana: Formal analysis, methodology, software, writing—original draft. Benjamin Harvey Mullish: Writing—review and editing. Michael Yee: Writing—review and editing. Elias Mossialos: Supervision, writing—review and editing. Robert Goldin: writing—review and editing. Salvatore Petta: writing—review and editing. Emmanouil Tsochatzis: writing - review and editing. Mark Thursz: conceptualization, supervision, writing—review and editing. Pinelopi Manousou: conceptualization, funding acquisition, supervision, writing - review and editing. All the authors have reviewed and approved the final draft.

FUNDING INFORMATION

RF is the recipient of the EASL PhD fellowship Juan Rodes 2018. BHM is the recipient of an NIHR Academic Clinical Lectureship (CL-2019-21-002). The Division of Digestive Diseases receives financial support from the National Institute of Health Research (NIHR) Imperial Biomedical Research Centre (BRC).

CONFLICT OF INTEREST STATEMENT

The authors do not have any disclosures to report.

DATA AVAILABILITY STATEMENT

All data and results from the study are included in the article.

ETHICAL APPROVAL STATEMENT

The study obtained full ethical approval from the Research Ethics Committee (REC approval 18/LO/1742, IRAS 251274).

PATIENT CONSENT STATEMENT

All the patients gave written consent to take part to the study.

ORCID

Roberta Forlano https://orcid.org/0000-0003-4746-7065
Benjamin Harvey Mullish https://orcid.org/0000-0001-6300-3100
Salvatore Petta https://orcid.org/0000-0002-0822-9673
Emmanouil Tsochatzis https://orcid.org/0000-0001-5069-2461

REFERENCES

1. Le MH, Yeo YH, Li X, et al. 2019 global NAFLD prevalence: a systematic review and meta-analysis. *Clin Gastroenterol Hepatol*. 2022;20(12):2809-2817 e28. doi:10.1016/j.cgh.2021.12.002
2. Paik JM, Golabi P, Younossi Y, Mishra A, Younossi ZM. Changes in the global burden of chronic liver diseases from 2012 to 2017: the growing impact of NAFLD. *Hepatology*. 2020;72(5):1605-1616. doi:10.1002/hep.31173
3. Kleiner DE, Makhlouf HR. Histology of nonalcoholic fatty liver disease and nonalcoholic Steatohepatitis in adults and children. *Clin Liver Dis*. 2016;20(2):293-312. doi:10.1016/j.cld.2015.10.011
4. Hagstrom H, Nasr P, Ekstedt M, et al. Fibrosis stage but not NASH predicts mortality and time to development of severe liver disease in biopsy-proven NAFLD. *J Hepatol*. 2017;67(6):1265-1273. doi:10.1016/j.jhep.2017.07.027
5. Mantovani A, Petracca G, Beatrice G, Tilg H, Byrne CD, Targher G. Non-alcoholic fatty liver disease and risk of incident diabetes mellitus: an updated meta-analysis of 501 022 adult individuals. *Gut*. 2021;70(5):962-969. doi:10.1136/gutjnl-2020-322572
6. Jarvis H, Craig D, Barker R, et al. Metabolic risk factors and incident advanced liver disease in non-alcoholic fatty liver disease (NAFLD): a systematic review and meta-analysis of population-based observational studies. *PLoS Med*. 2020;17(4):e1003100. doi:10.1371/journal.pmed.1003100
7. Younossi ZM, Golabi P, Paik JM, Henry A, Van Dongen C, Henry L. The global epidemiology of nonalcoholic fatty liver disease (NAFLD) and nonalcoholic steatohepatitis (NASH): a systematic review. *Hepatology*. 2023;77:1335-1347. doi:10.1097/HEP.0000000000000004
8. Noureddin M, Jones C, Alkhouri N, et al. Screening for nonalcoholic fatty liver disease in persons with type 2 diabetes in the United States is cost-effective: a comprehensive cost-utility analysis. *Gastroenterology*. 2020;159(5):1985-1987 e4. doi:10.1053/j.gastro.2020.07.050
9. Sheridan DA, Aithal G, Alazawi W, et al. Care standards for non-alcoholic fatty liver disease in the United Kingdom 2016: a cross-sectional survey. *Frontline Gastroenterol*. 2017;8(4):252-259. doi:10.1136/flgastro-2017-100806
10. Rinella ME, Neuschwander-Tetri BA, Siddiqui MS, et al. AASLD practice guidance on the clinical assessment and management of

14783231, 2024, 1, Downloaded from https://onlinelibrary.wiley.com/doi/10.1111/liv.15730 by Cochrane Saudi Arabia, Wiley Online Library on [20/05/2024]. See the Terms and Conditions (https://onlinelibrary.wiley.com/terms-and-conditions) on Wiley Online Library for rules of use; OA articles are governed by the applicable Creative Commons License

nonalcoholic fatty liver disease. *Hepatology*. 2023;77(5):1797-1835. doi:10.1097/HEP.0000000000000323
11. European Association for the Study of the Liver. Electronic address eee, clinical practice guideline P, chair, representative EGB, panel m. EASL clinical practice guidelines on non-invasive tests for evaluation of liver disease severity and prognosis—2021 update. *J Hepatol* Sep 2021;75(3):659–689. doi:10.1016/j.jhep.2021.05.025
12. Mansour D, Grapes A, Herscovitz M, et al. Embedding assessment of liver fibrosis into routine diabetic review in primary care. *JHEP Rep*. 2021;3(4):100293. doi:10.1016/j.jhepr.2021.100293
13. ElSayed NA, Aleppo G, Aroda VR, et al. 4. Comprehensive medical evaluation and assessment of comorbidities: standards of care in diabetes-2023. *Diabetes Care*. 2023;46(suppl 1):S49-S67. doi:10.2337/dc23-S004
14. Dobbie LJ, Kassab M, Davison AS, Grace P, Cuthbertson DJ, Hydes TJ. Low screening rates despite a high prevalence of significant liver fibrosis in people with diabetes from primary and secondary care. *J Clin Med* Dec 9 2021;10(24):5755. doi:10.3390/jcm10245755
15. Tanajewski L, Harris R, Harman DJ, et al. Economic evaluation of a community-based diagnostic pathway to stratify adults for non-alcoholic fatty liver disease: a Markov model informed by a feasibility study. *BMJ Open*. 2017;7(6):e015659. doi:10.1136/bmjopen-2016-015659
16. Thavorn K, Coyle D. Transient Elastography and controlled attenuation parameter for diagnosing liver fibrosis and steatosis in Ontario: an economic analysis. *Ont Health Technol Assess Ser.* 2015;15(19):1-58.
17. Srivastava A, Jong S, Gola A, et al. Cost-comparison analysis of FIB-4, ELF and fibroscan in community pathways for non-alcoholic fatty liver disease. *BMC Gastroenterol*. 2019;19(1):122. doi:10.1186/s12876-019-1039-4
18. Guide to the methods of technology appraisal. 2013.
19. The guidelines manual. 2012 (2013).
20. Williams R, Aspinall R, Bellis M, et al. Addressing liver disease in the UK: a blueprint for attaining excellence in health care and reducing premature mortality from lifestyle issues of excess consumption of alcohol, obesity, and viral hepatitis. *Lancet*. 2014;384(9958):1953-1997. doi:10.1016/S0140-6736(14)61838-9
21. Lazarus JV, Mark HE, Anstee QM, et al. Advancing the global public health agenda for NAFLD: a consensus statement. *Nat Rev Gastroenterol Hepatol*. 2022;19(1):60-78. doi:10.1038/s41575-021-00523-4
22. Kanwal F, Kramer JR, Li L, et al. Effect of metabolic traits on the risk of cirrhosis and hepatocellular cancer in nonalcoholic fatty liver disease. *Hepatology*. 2020;71(3):808-819. doi:10.1002/hep.31014
23. Ajmera V, Cepin S, Tesfai K, et al. A prospective study on the prevalence of NAFLD, advanced fibrosis, cirrhosis and hepatocellular carcinoma in people with type 2 diabetes. *J Hepatol*. 2022;78:471-478. doi:10.1016/j.jhep.2022.11.010
24. Stroffolini T, Sagnelli E, Sagnelli C, et al. The association between education level and chronic liver disease of any etiology. *Eur J Intern Med*. 2020;75:55-59. doi:10.1016/j.ejim.2020.01.008
25. Blais P, Husain N, Kramer JR, Kowalkowski M, El-Serag H, Kanwal F. Nonalcoholic fatty liver disease is underrecognized in the primary care setting. *Am J Gastroenterol*. 2015;110(1):10-14. doi:10.1038/ajg.2014.134
26. Forlano R, Mullish BH, Dhar A, Goldin RD, Thursz M, Manousou P. Liver function tests and metabolic-associated fatty liver disease: changes in upper normal limits, does it really matter? *World J Hepatol*. 2021;13(12):2104-2112. doi:10.4254/wjh.v13.i12.2104
27. Graupera I, Thiele M, Serra-Burriel M, Caballeria L, Roulot D, Wong GL, Fabrellas N, Guha IN, Arslanow A, Expósito C, Hernández R, Aithal GP, Galle PR, Pera G, Wong VW, Lammert F, Ginès P, Castera L, Krag A, Investigators of the LiverScreen Consortium Low accuracy of FIB-4 and NAFLD fibrosis scores for screening for liver fibrosis in the population. *Clin Gastroenterol Hepatol* Nov 2022;20(11):2567–2576 e6. doi:10.1016/j.cgh.2021.12.034
28. Eng P, Chia FR, Tan T, Manousou P, Dhillo WS, Izzi-Engbeaya C. Non-alcoholic fatty liver disease in women – current knowledge and emerging concepts. *JHEP Rep*. 2023;100835. doi:10.1016/j.jhepr.2023.100835
29. Boursier J, Canivet CM, Costentin C, et al. Impact of type 2 diabetes on the accuracy of noninvasive tests of liver fibrosis with resulting clinical implications. *Clin Gastroenterol Hepatol*. 2023;21(5):1243-1251 e12. doi:10.1016/j.cgh.2022.02.059
30. Lassailly G, Caiazzo R, Ntandja-Wandji LC, et al. Bariatric surgery provides Long-term resolution of nonalcoholic Steatohepatitis and regression of fibrosis. *Gastroenterology*. 2020;159(4):1290-1301 e5. doi:10.1053/j.gastro.2020.06.006
31. European Association for the Study of the L, European Association for the Study of D, European Association for the Study of O. EASL-EASD-EASO clinical practice guidelines for the management of non-alcoholic fatty liver disease. *J Hepatol*. 2016;64(6):1388-1402. doi:10.1016/j.jhep.2015.11.004
32. Mantovani A, Csermely A, Petracca G, et al. Non-alcoholic fatty liver disease and risk of fatal and non-fatal cardiovascular events: an updated systematic review and meta-analysis. *Lancet Gastroenterol Hepatol*. 2021;6(11):903-913. doi:10.1016/S2468-1253(21)00308-3
33. Serra-Burriel M, Graupera I, Toran P, et al. Transient elastography for screening of liver fibrosis: cost-effectiveness analysis from six prospective cohorts in Europe and Asia. *J Hepatol*. 2019;71(6):1141-1151. doi:10.1016/j.jhep.2019.08.019
34. Tapper EB, Hunink MG, Afdhal NH, Lai M, Sengupta N. Cost-effectiveness analysis: risk stratification of nonalcoholic fatty liver disease (NAFLD) by the primary care physician using the NAFLD fibrosis score. *PloS One*. 2016;11(2):e0147237. doi:10.1371/journal.pone.0147237
35. Kim BK, Tamaki N, Imajo K, et al. Head-to-head comparison between MEFIB, MAST, and FAST for detecting stage 2 fibrosis or higher among patients with NAFLD. *J Hepatol*. 2022;77(6):1482-1490. doi:10.1016/j.jhep.2022.07.020
36. Anstee QM, Castera L, Loomba R. Impact of non-invasive biomarkers on hepatology practice: past, present and future. *J Hepatol*. 2022;76(6):1362-1378. doi:10.1016/j.jhep.2022.03.026

SUPPORTING INFORMATION

Additional supporting information can be found online in the Supporting Information section at the end of this article.

How to cite this article: Forlano R, Stanic T, Jayawardana S, et al. A prospective study on the prevalence of MASLD in people with type-2 diabetes in the community. Cost effectiveness of screening strategies. *Liver Int*. 2024;44:61-71. doi:10.1111/liv.15730

14783231, 2024, 1, Downloaded from https://onlinelibrary.wiley.com/doi/10.1111/liv.15730 by Cochrane Saudi Arabia, Wiley Online Library on [20/05/2024]. See the Terms and Conditions (https://onlinelibrary.wiley.com/terms-and-conditions) on Wiley Online Library for rules of use; OA articles are governed by the applicable Creative Commons License
